# Allopregnanolone Preclinical Acute Pharmacokinetic and Pharmacodynamic Studies to Predict Tolerability and Efficacy for Alzheimer’s Disease

**DOI:** 10.1371/journal.pone.0128313

**Published:** 2015-06-03

**Authors:** Ronald W. Irwin, Christine M. Solinsky, Carlos M. Loya, Francesco G. Salituro, Kathleen E. Rodgers, Gerhard Bauer, Michael A. Rogawski, Roberta Diaz Brinton

**Affiliations:** 1 Department of Pharmacology and Pharmaceutical Science, School of Pharmacy, University of Southern California, Los Angeles, California, United States of America; 2 Clinical and Experimental Therapeutics Program, University of Southern California, Los Angeles, California, United States of America; 3 Sage Therapeutics, Cambridge, Massachusetts, United States of America; 4 Titus Family Department of Clinical Pharmacy and Pharmaceutical Economics & Policy, School of Pharmacy, University of Southern California, Los Angeles, California, United States of America; 5 Department of Internal Medicine, School of Medicine, University of California Davis, Sacramento, California, United States of America; 6 Department of Neurology, School of Medicine, University of California Davis, Sacramento, California, United States of America; 7 Department of Neurology, Keck School of Medicine, University of Southern California Los Angeles, Los Angeles, California, United states of America; Rosalind Franklin University, UNITED STATES

## Abstract

To develop allopregnanolone as a therapeutic for Alzheimer’s disease, we investigated multiple formulations and routes of administration in translationally relevant animal models of both sexes. Subcutaneous, topical (transdermal and intranasal), intramuscular, and intravenous allopregnanolone were bolus-administered. Pharmacokinetic analyses of intravenous allopregnanolone in rabbit and mouse indicated that peak plasma and brain levels (3-fold brain/plasma ratios) at 5min were sufficient to activate neuroregenerative responses at sub-sedative doses. Slow-release subcutaneous suspension of allopregnanolone displayed 5-fold brain/plasma ratio at C_max_ at 30min. At therapeutic doses by either subcutaneous or intravenous routes, allopregnanolone mouse plasma levels ranged between 34-51ng/ml by 30min, comparable to published endogenous human level in the third trimester of pregnancy. Exposure to subcutaneous, topical, intramuscular, and intravenous allopregnanolone, at safe and tolerable doses, increased hippocampal markers of neurogenesis including BrdU and PCNA in young 3xTgAD and aged wildtype mice. Intravenous allopregnanolone transiently and robustly phosphorylated CREB within 5min and increased levels of neuronal differentiation transcription factor NeuroD within 4h. Neurogenic efficacy was achieved with allopregnanolone brain exposure of 300-500hr*ng/g. Formulations were tested to determine the no observable adverse effect level (NOAEL) and maximally tolerated doses (MTD) in male and female rats by sedation behavior time course. Sex differences were apparent, males exhibited ≥40% more sedation time compared to females. Allopregnanolone formulated in sulfobutyl-ether-beta-cyclodextrin at optimized complexation ratio maximized allopregnanolone delivery and neurogenic efficacy. To establish the NOAEL and MTD for Allo-induced sedation using a once-per-week intravenous regenerative treatment regimen: In female rats the NOAEL was 0.5mg/kg and MTD 2mg/kg. The predicted MTD in human female is 0.37mg/kg. In male rats the NOAEL and MTD were less than those determined for female. Outcomes of these PK/PD studies predict a safe and efficacious dose range for initial clinical trials of allopregnanolone for Alzheimer’s disease. These findings have translational relevance to multiple neurodegenerative conditions.

## Introduction

To date, no therapeutic intervention exists to prevent, delay, or treat Alzheimer's disease [1, 2]. Recent failed Phase 3 trials targeting beta-amyloid plaques are indicative of the complexity of the multifactorial disease process and highlight the need for alternative innovative therapeutics [3, 4]. A novel therapeutic approach targets the regenerative neurogenic capacity of the brain to sustain neurological function and to prevent, delay or treat neurodegenerative diseases [5]. In adults, the subgranular zone of the hippocampus dentate gyrus and the subventricular zone of the lateral ventricle comprise the two most prolific neurogenic niches [6]. Multiple studies indicate that adult human neurogenesis occurs and is sustained throughout the lifespan in the disease-free brain [7–9]. Previously, we demonstrated that the neurosteroid allopregnanolone (Allo) promotes neurogenesis *in vivo* [10] and proliferation of rodent and human neural progenitor cells *in vitro* [11]. Allo increased neurogenesis within the hippocampus and restored learning and memory function to normal prior to and following the onset of Alzheimer's disease pathology in the triple transgenic Alzheimer’s disease (3xTgAD) mouse [10, 12, 13]. Further, Allo was comparably efficacious in the aged wildtype mouse [13]. In 3xTgAD mice, Allo increased markers of white matter regeneration and cholesterol homeostasis while simultaneously reducing beta-amyloid burden and microglia inflammatory markers [12].

An optimal Allo dosing regimen of once per week significantly increased neurogenesis while simultaneously reducing Alzheimer’s related pathology [5, 12, 14]. Allo fulfills multiple requirements for drugs targeting the brain including a small molecular weight (318.49 g/mol); low number of hydrogen bond donors (one) and acceptors (two). The logP 5.042 value for Allo, poses a solubility challenge for aqueous formulation and thus makes formulation for oral administration difficult [15]. Parenteral (non-oral) routes of Allo administration are advantageous because they minimize first-pass metabolism through the liver. Allo is blood brain barrier penetrant molecule with previous safety data in humans [16–21]. A regenerative therapeutic regimen of once per week Allo increases the margin of safety by allowing clearance and recovery of the neuro-regenerative system prior to the next dose.

The mechanism of action for Allo activates cell cycle gene expression in neural stem cells via GABA_A_ receptor mediated chloride efflux. Allo potentiates the GABA-mediated chloride ion flux through GABA_A_ receptors resulting in depolarization of the plasma membrane to activate L-type voltage-dependent calcium channels followed by a rise in intracellular calcium and subsequent activation of the cell cycle [5, 11, 22]. The rapid time scale of Allo-activated signaling cascades suggested that injectable administration, wherein maximal blood and brain concentrations of Allo would be rapidly achieved and cleared, should be sufficient to activate the regenerative pathways required for Allo-induced neurogenesis [5].

Our preclinical data continue to indicate that the regenerative effect of Allo occurs optimally at sub-sedative doses in a regimen consistent with the time course for regeneration, i.e. a once per week exposure and not daily delivery as is typical for most therapeutics. The aim of the current analyses was to bridge previous Allo subcutaneous suspension delivery studies [10, 13] to soluble cyclodextrin-based formulations necessary for clinical translation. Formulations of Allo were tested by multiple routes of administration through a series of pharmacokinetic (PK) and dose range-finding studies to evaluate efficacy and tolerability parameters. Results of the current analyses indicated that by multiple routes, Allo rapidly promotes the regenerative capacity of the brain. This translational research is important for Alzheimer’s disease and other neurological disorders including Parkinson’s disease, multiple sclerosis, Niemann-Pick, Fragile-X syndrome, diabetic neuropathy, status epilepticus, and traumatic brain injury [2, 5].

## Materials and Methods

### Chemicals

All chemicals were from Sigma (St. Louis, MO) unless otherwise noted. Allopregnanolone for the rabbit pharmacokinetics study was purchased from Steraloids, Inc. (Newport, RI) and for all other studies was provided by Dr. M.A. Rogawski (University of California, Davis). (2-hydroxypropyl)-beta-cyclodextrin (HBCD) was obtained from Cyclodextrin Technologies Development, Inc. (High Springs, FL). Sulfobutylether-beta-cyclodextrin (SBECD) obtained from CycloLab Cyclodextrin Research and Development Laboratory, Ltd (Budapest, Hungary) was used for the intramuscular mouse efficacy study. SBECD obtained from CyDex, Pharmaceuticals, Inc. (Lenexa, KS) was used for all other SBECD containing studies.

### Animals

#### Ethical Treatment of Animals

All rodent experiments were performed following National Institutes of Health guidelines on ethical care and use of laboratory animals and protocols specifically approved for this study by the Institutional Animal Care and Use Committee of the University of Southern California (Los Angeles, CA) (Protocol numbers: 11156 and 20127). All procedures in rabbits were conducted at SRI, International (Menlo Park, CA) and specifically approved for this study by the SRI Institutional Animal Care and Use Committee in accordance with the National Research Council (NRC) Guide for the Care and Use of Laboratory Animals (1996), and the Animal Welfare Standards incorporated in 9 CFR Part 3, 1991. All animals were housed with food and water *ad libitum* under a 12-h light/dark cycle. All procedures were performed with strict adherence to protocol and all efforts were made to minimize suffering.

#### Rabbits

Adult, female New Zealand White rabbits (5–14 months, 3.6–4.5 kg) were purchased from Harlan (Indianapolis, IN). Each rabbit was individually housed in the animal facility at SRI, International (Menlo Park, CA) under standard conditions and cared for in accordance with SRI Institutional Animal Care and Use Committee, the National Research Council (NRC) *Guide for the Care and Use of Laboratory Animals* (1996), and the Animal Welfare Standards incorporated in 9 CFR Part 3, 1991.

#### Transgenic mice

Colonies of triple-transgenic Alzheimer’s mouse model (3xTgAD) and their background strain nontransgenic wildtype mouse (C57BL/6/129S; a gift from Dr. F.M. LaFerla, University of California, Irvine) [23] were bred and maintained at the University of Southern California (Los Angeles, CA) following National Institutes of Health guidelines on use of laboratory animals and an approved protocol for this study by the University of Southern California Institutional Animal Care and Use Committee (Protocol Number: 11156). Mice were group-housed 2–5 per cage on 12-h light/dark cycles and provided *ad libitum* access to food and water. The 3xTgAD mouse model was created by a genetic knock-in of presenilin-1 to single-cell mouse embryos with the PS1M146V mutation co-injected with two human transgenes (APP with the Swedish mutation and Tau with the P301L mutation) [23]. The gene mutations (human APP_SWE_, Tau_P301L_, and PS1_M146V_) are linked to AD and fronto-temporal dementia and exhibits an age-related neuropathological phenotype including both beta-amyloid deposition and tau hyperphosphorylation [23]. Translation of the overexpressed transgenes is primarily restricted to the central nervous system, notably in the cerebral cortex, amygdala, and hippocampus. Transgenes integrated at a single locus under the control of the mouse Thy1.2 promoter and these mice are homozygous, viable, fertile and display no initial gross physical or behavioral abnormalities. To ensure the stability of AD-like phenotype in the 3xTgAD mouse colony, we performed routine immunohistochemical assays every 3 to 4 generations. Only offspring from breeders that exhibited stable AD pathology were randomized into the study. In parallel with the 3xTgAD mouse model, we tested the wildtype or nontransgenic (WT; nonTg) 129SvB6 background strain animals at 15 months of age as a model of aging. We found previously 3xTgAD mice younger than 12 months of age and nonTg mice at 15 months of age were responsive to Allo, determined by a hippocampal-dependent associative learning and memory task and survival of BrdU-labeled hippocampal cells [13].

#### Rats

Male and female Sprague-Dawley rats were purchased from Harlan (Indianapolis, IN) following National Institutes of Health guidelines on use of laboratory animals and an approved protocol for this study by the University of Southern California Institutional Animal Care and Use Committee (Protocol Number: 20127). Each rat was individually housed in the animal facilities at the University of Southern California (Los Angeles, CA). Rats were housed on 12 h light/dark cycles and provided *ad libitum* access to food and water. All rats received from Harlan were proven breeders and females were ovariectomized prior to shipment at 6 months of age. As a reference, endogenous Allo blood and brain levels in gonadally-intact females have been reported 1.6 ng/g Allo [24]. Seven days after surgical removal of ovaries, the level of Allo in blood plasma has been reported to decrease to 0.2 ng/g, a level similar to male rats, and within 4 months of ovariectomy Allo blood levels only slightly increased to 0.33 ng/g [24]. Endogenous rat brain cortex Allo was previously reported at 4.19 ng/g prior to ovariectomy and that level decreased to 2.46 ng/g, a level similar to males, within 4 months of ovariectomy [24]. Upon arrival, all rats were acclimated for at least two weeks prior to behavioral test. At initiation of Allo dose administration, all rats were age-matched with mean age: 7.12 ± 0.15 months old and body weight: male 483 ± 30 g and ovariectomized female 318 ± 19 g. After completion of all behavioral testing with intermittent dosing by multiple routes, rats were euthanized with 4% isoflurane anesthesia inhalant followed by dissection, with mean age 11.12 ± 0.15 months old and body weight: male 533 ± 34 g and female 331 ± 17 g. Low uterine weight confirmed ovariectomy procedure: 0.158 ± 0.028 g. Brain weight was recorded: male 1.88 ± 0.07 g and female 1.67 ± 0.07 g. No gross abnormalities in organs were observed and all rats survived to study completion with no clinical symptoms.

### Drug preparation

#### Rabbit IV pharmacokinetics study

Allo was dissolved in 20%w/v HBCD solution at 1.5 mg/ml by brief sonication. The pH was recorded as 7.1. The formulation was filter sterilized using a 0.2 μm filter (Millipore, Billerica, MA, USA).

#### Rabbit TD pharmacokinetics study

Allo was added to dimethyl sulfoxide (DMSO; Mallinckrodt, Phillipsburg, NJ), 20 mg/ml, and vigorously mixed on a vortex mixer and sonicated until Allo was visibly dissolved.

#### Mouse pharmacokinetics and efficacy studies

Allo was dissolved in 6%w/v HBCD solution at 0.5 mg/ml by brief sonication and was administered intravenously (IV) to mice at 1.5 mg/kg via lateral tail vein. Allo was dissolved in 20%w/v HBCD solution at 2.5 mg/ml by brief sonication and was subcutaneously (SC) injected to mice at 0.5, 1, and 10 mg/kg. Additionally, Allo was dissolved in 6%w/v SBECD solution at 0.5 mg/ml and injected IV to mice at 0.1, 0.5, and 1 mg/kg. HBCD or SBECD alone were included as vehicle controls. Topical transdermal (TD) was applied on the shaved dorsal surface at 50mg/kg using a gel solution of 3.3% Allo (w/w), 45% DMSO, 30% EtOH, 2.5% Klucel MF, 19.2% PEG-300. Intranasal (IN) formulations were prepared in both 100% castor oil and 20% HBCD. Intramuscular (IM) formulation was administered to mice as Allo 1.5 mg/ml in 6% SBECD. As a positive control to our previous studies, SC 10 mg/kg 2.5 mg/ml; PBS/5%EtOH was administered as a suspension formulation. For 24 h cell proliferation studies, the thymidine analogue, 5-Bromo-2’-deoxyuridine (BrdU), incorporated into newly synthesized DNA of replicating cells during the S-Phase of the cell cycle, was dissolved in PBS and intraperitoneally injected at 100 mg/kg 1 h following Allo treatment.

#### Rat sedation/formulations study

Allo was dissolved in 6%w/v SBECD at 1.5 mg/ml by brief sonication and administered IV to rats at 0.5–2 mg/kg. Additionally, Allo was dissolved in 24%w/v SBECD at 6 mg/ml, 1.5 mg/ml and 24 mg/ml by brief sonication and administered by SC and intramuscular (IM) routes to the rats in doses ranging from 2–8 mg/kg. As a control comparison to our previous Allo efficacy studies [10, 13], Allo 2.5 mg/ml dissolved in ethanol, was diluted to 5% solution 95% phosphate buffered saline, administered as a suspension SC to rats at 8 mg/kg and a consistent sedation score of 4, no detectable sedation, was obtained (*n* = 4; data not shown). A serial dilution test in 24% SBECD, found that Allo reaches a saturation point between 8–10 mg/ml coinciding with a molar ratio of 4–5 SBECD molecules per Allo molecule in water at room temperature without pH adjustment [2].

### Pharmacokinetic studies

#### Rabbits

Rabbit studies were conducted and analyzed by SRI, International (Menlo Park, CA), a contract research organization. Rabbits were randomly assigned to treatment groups by a computerized body weight stratification procedure (Labcat In-Life version 8.0 SP2). Blood was collected from ear vessel into tubes containing EDTA, processed to plasma, and then stored frozen at ≤-60°C. Blood was not collected from the vein in which drug was administered. Rabbits were not fasted prior to blood collection. After administration of IV Allo 3 mg/kg and TD Allo ~5 mg/kg, blood samples were collected pre-dose, and at 5, 15, 30 minutes, and 1, 2, 4, 8, 24 hours post-dose, or until sacrifice. For dermal topical administration, a ~10 cm x10 cm interscapular area on the back of each rabbit was clipped free of fur where the topical formulation was to be applied. Animals were sacrificed through an overdose of sodium pentobarbital administered by IV injection. The brain was collected at necropsy (excluding olfactory bulbs), cut sagittally through the midline into two halves. The brain sections were immediately snap-frozen in liquid nitrogen. Rabbits were euthanized for brain collection at a variety of time points after Allo administration to facilitate brain sample collection (IV: 5 min, *n* = 1; 30 min, *n* = 3; 4 h, *n* = 3; 24 h, *n* = 2 and TD: 30 min, *n* = 3; 4 h, *n* = 3; 24 h, *n* = 3). IV-treated rabbits were sedated and non-responsive for 20–30 min after dose administration. One rabbit treated with IV Allo 3 mg/kg bolus did not recover and died at 5 min after dose administration and its brain tissue was collected. The remaining rabbits survived and did not show clinical symptoms.

On Day 1, topically administered Allo formulated in 100% DMSO caused moderate to severe erythema/edema and moist weeping on the skin in 8 of 9 rabbits. On Day 2 (final study day), the symptoms had not resolved in 6 rabbits. These observations are typical of topically applied DMSO. No mortality occurred in this group with topical delivery. Individual animal plasma and brain sample analysis and quantification of Allo was done using LC-MS/MS. Pharmacokinetic (PK) parameters were determined using noncompartmental methods with the sparse sampling option, which provides a group analysis of limited plasma data from multiple animals. Plasma and brain concentration were included if above the lower limit of quantitation (LLOQ; Allo 2 ng/ml (6.28 nanomolar) plasma; Allo 10 ng/g brain) of the bioanalytical assay before the first measurable concentration were assigned a value of 1 in the calculation of mean concentrations and PK parameters. Allo brain levels were reported as ng per g of brain tissue (ng/g). LLOQ of 2 ng/ml in the assay of brain homogenates corresponds to an Allo concentration of 10.01 ± 0.27 ng/g brain tissue. The Allo concentrations were obtained and data input to Excel 2010 (Microsoft, Redmond, WA) Data Analysis Toolpak, to calculate PK parameters using a computer-assisted method (PK functions for Microsoft Excel; J. L. Usansky, A. Desai, and D. Tang-Liu, Department of Pharmacokinetics and Drug Metabolism, Allergan, Irvine, CA [http://www.boomer.org/pkin/soft.html]). PK data analysis was performed using a uniform weighting scheme, and AUC was calculated using the linear up/log down trapezoidal method. To calculate t_1/2_, time points from the terminal elimination phase were selected manually, with a minimum of three non-zero plasma concentration values after the T_max_ required.

#### Bioanalytical Method

Rabbit plasma and brain samples were analyzed by LC-MS/MS (SRI, International Menlo Park, CA). Rabbit blood plasma (100 μl) and d4-Allo (500 ng/ml in water; (100 μl)) were combined with 1000 μl tert-butyl methyl ether to precipitate proteins. Brain samples were prepared by addition of 4 volumes of phosphate-buffered saline to a pre-weighed brain in a 15 ml Falcon tube. The brain tissue was homogenized on ice using a Polytron tissue grinder. Duplicate 100 μl aliquots of the resulting homogenates were processed exactly as described for the preparation of plasma samples. Both plasma and brain samples were vortexed for 10 min on a multi-tube vortex mixer at maximal speed followed by centrifugation (18000 x g, 5 min). The supernatant (800 μl) were transferred to a 1.5 ml microfuge tube, and dried under vacuum centrifugation. The samples were reconstituted with 50 μl of methanol and briefly vortexed on a multi-tube vortex mixer at maximal speed. Phenyl hydrazine-HCl (10mM; 50 μl) was added to form the phenylhydrazone of Allo then briefly vortexed and incubated in the dark for 6 h. Samples were transferred to HPLC vials fitted with glass inserts for LC-MS/MS analysis. Study samples were quantitated using a set of calibration standards prepared in blank matrix that were processed in parallel. Samples were injected onto a Waters Aquity UPLC System with Luna C8(2) column (50 × 2.00 mm; 5 μm; Phenomenex, Torrance, CA) with a mobile phase A (0.1% formic acid in water) and mobile phase B (0.1% formic acid in acetonitrile) and eluted at a flow rate of 0.35 ml/min with the following gradient conditions: 0 min 20% A and 80% B; 6 min 50% A and 50% B. Quantification was performed using a Micromass Quattro Micro Mass Spectrometer (Waters, Milford, MA) operating in positive ion mode monitoring the multiple-reaction ion transition of m/z 409.2 to m/z 161.2 (plasma) or m/z 409.3 to m/z 160.9 (brain). The desolvation temperature was 375°C, the capillary voltage was 1.25 kV, the cone voltage was 40 V, and the collision energy was -30 eV (plasma) or -25 eV (brain). Quantitation of Allo in plasma samples using calibration standards was as follows: Peak areas of the analyte (Allo) were divided by the peak area of d4-Allo (internal standard; C/D/N/ Isotopes, Inc., Point Claire, Quebec, Canada) to yield peak area ratios. The calibration standard curve for Allo was prepared by performing weighted linear regression (1/y) of the peak area ratio of Allo as the dependent variable (y-axis) and concentration as the independent variable (x-axis). Integration and quantitation was performed by the Quanlynx portion of Masslynx Software ver. 4.1 for plasma and 4.0 for brain.

#### Mice

Male mice were randomly assigned to treatment groups by an age and body weight stratification procedure. After administration of IV Allo 1.5 mg/kg, blood samples were collected until sacrifice at pre-dose, 5, 15, 30 min, 4 h and 24 h post-dose. Blood samples were collected pre-dose and after administration of SC Allo 10mg/kg, at 30 min, 4 h, and 24 h post-dose or until sacrifice. Mice were not fasted prior to blood collection. Mice were anesthetized with 100 mg/kg ketamine and 10 mg/kg xylazine and euthanized by cervical dislocation. Trunk blood was collected into tubes containing EDTA. Brains were immediately collected and dissected along the sagittal line into two hemispheres; the left hemisphere was snap frozen on dry ice and then stored at -80°C for biochemical analysis. Mice were euthanized for brain collection at multiple timepoints after Allo administration (IV: 5 min, 15min, 30 min, 4 h, 24 h, all n = 5–6) (SC: 30 min, 4 h, 24 h, all n = 3). Studies were conducted at the University of Southern California and animal plasma and brain sample analysis and quantification of Allo was contracted to Agilux Laboratories (Worcester, MA) and conducted by LC-MS/MS. Brain samples were homogenized in 80:20 water:acetonitrile (4:1 w:v) and analyzed against a brain tissue standard curve. The Allo concentrations were obtained and data input to Excel 2010 (Microsoft, Redmond, WA) Data Analysis Toolpak, to calculate PK parameters using a computer-assisted method (PK functions for Microsoft Excel; J. L. Usansky, A. Desai, and D. Tang-Liu, Department of Pharmacokinetics and Drug Metabolism, Allergan, Irvine, CA [http://www.boomer.org/pkin/soft.html]). Allo brain levels were reported as ng per g of brain tissue (ng/g). As a reference point, endogenous Allo levels have been previously measured in brain to be ~2.25 ng/g in young wildtype mice and this level decreases to ~1.25 ng/g in wildtype 24-month male mice. In 3xTgAD male mice the brain levels range from 1.8–1.9 ng/g [25].

#### Bioanalytical Method

Mouse plasma and brain samples were processed by liquid-liquid extraction and the supernatants were analyzed by LC-MS/MS (Agilux Laboratories, Worcester, MA). Samples were injected onto a Waters Aquity UPLC System with BEH C18 column (2.1 x 50mm; 1.7 μm) with a mobile phase A (95:5:0.1 H2O:ACN:FA) and mobile phase B (50:50:0.1 MeOH:ACN:FA) and eluted at a flow rate of 0.5 ml/min with the following gradient conditions: 0 min 70% A and 30% B; 0.2 min 70% A and 30% B; 1.6 min 35% A and 65% B; 3.2 min 15% A and 85% B; 3.5 min 15% A and 85% B; 3.6 min 70% A and 30% B; 4 min 70% A and 30% B. Quantification was performed using a API 5500 Mass Spectrometer (AB SCIEX, Framingham, MA) operating in positive ion mode monitoring the multiple-reaction ion transition of m/z 319.1 to m/z 283.3. The desolvation temperature was 550°C. Dose forms were diluted 1000X (10X in DMSO followed by another 100X dilution into mouse plasma) and analyzed against a matrix-matched calibration curve. Calculated concentrations for dose formulations were within +15% of nominal in all cases with %CV for the three replicates <10%. Quantitation of Allo in plasma samples using calibration standards was as follows: Peak areas of the analyte (Allo) were divided by the peak area of testosterone (internal standard) to yield peak area ratios. The calibration standard curve for Allo was prepared by performing weighted linear regression (1/x^2^) of the peak area ratio of Allo as the dependent variable (y-axis) and concentration as the independent variable (x-axis).

### Pharmacokinetic parameters calculated

#### Rabbits

The following plasma PK parameters were reported for Allo: maximal plasma concentration after intravenous administration (C_max_), plasma concentration at the first sampling time point after IV administration (C_p_), time to reach C_max_ or C_p_ (T_max_), area under the plasma concentration time curve (AUC) up to the last sampling time point (AUC_last_), AUC extrapolated to infinity (AUC_inf_), mean residence time up to the last sampling time point (MRT_last_), terminal elimination half-life (t_1/2_), apparent volume of distribution (V), and total clearance (Cl). Allo exposure in brain was estimated by determining C_max_, T_max_, and AUC_last_ from Allo brain levels. Comparisons of brain-to-plasma exposure for each dose route were calculated as ratios of C_max_ and AUC_last_.

#### Mice

PK parameters reported for Allo: maximal plasma concentration after intravenous administration (Cmax), time to reach Cmax or Cp (T_max_), area under the plasma concentration time curve (AUC) up to the last sampling time point (AUC_last_), AUC extrapolated to infinity (AUC_inf_), terminal elimination half-life (t_1/2z_), and elimination constant (k). Allo exposure in brain was estimated by determining *C*_*max*_, *T*_*max*_, and *AUC*_*last*_ from Allo brain levels. Comparisons of brain-to-plasma exposure for each dose route were calculated as ratios of *C*_*max*_ and *AUC*_*last*_.

### Western blot analysis

All comparisons were made within blots. Equal amounts of protein (20–40 μg depending on the protein of interest) were separated by SDS-PAGE on a 12% or 10–20% Bis-Tris gel (Bio-Rad), transferred to 0.45 μm PVDF membrane (Millipore Corp., Bedford, MA). Nonspecific binding sites were blocked with blocking buffer (5% nonfat milk in Tris-buffered saline, TBS, containing 0.1% Tween-20, TBST). After blocking, primary antibodies were incubated with membrane in blocking buffer overnight at 4°C. The following antibodies were used in this study: NeuroD (1:1000, Cell Signaling Technology), Doublecortin (1:300, AbCam), proliferating cell nuclear antigen (PCNA; 1:1000, Millipore), CyclinD2 (1:1000, Cell Signaling Technology), pCREB (1:1000, Cell Signaling Technology), CREB (1:1000, Cell Signaling Technology). The membranes were then incubated with a horseradish peroxidase-conjugated anti-rabbit antibody or anti-mouse secondary antibody (1:5000–1:10,000) complementary to the primary antibody. Immunoreactive bands were visualized by Pierce SuperSignal Chemiluminescent Substrates (Thermo Scientific) and captured by Molecular Imager ChemiDoc XRS System (Bio-Rad Laboratories). All band intensities were quantified using Un-Scan-it software. Protein expression was normalized by loading house keeping protein β-actin (1:6000, Millipore). It is not possible to determine from whole hippocampus protein lysates, whether or not the proteins associated with cell cycle, proliferation, or differentiation *in vivo* are co-expressed or differentially expressed within each cell, cell type, or sub-hippocampal region.

### Flow-cytometry

In order to reduce the labor-intensive and time-consuming demands of using immunohistochemistry combined with unbiased stereology to analyze the Allo-induced formation of new cells in hippocampus of 6-, 9-, 12-, and 15-month-old mice, we chose to assess the BrdU immunopositive nuclei using flow cytometry assay, which we have demonstrated to more objectively screen a large amount of samples in a high-throughput manner to evaluate the efficacy of a neurogenic agent versus immunohistochemistry combined with unbiased stereology [10, 13, 26]. Hippocampi were dissected from the fixed hemispheres from the cell survival assessment experiment using consistent anatomical landmarks as criteria for dissection as described before [27]. The fornix adjacent to the hippocampal lobe was removed to avoid the subventricular zone and rostral migratory stream proliferative pools. The extracted hippocampi were homogenized using Next Advance 24-sample homogenizer (Next Advance, Inc., Averill Park, NY, USA) for 3 minutes on speed 7. This procedure lyses the plasma lemma while preserving the integrity of the nuclear envelope. The nuclei were collected into a 1.5 mL microcentrifuge tube by washing the beads and tube 4 times using 200 μL of PBS, and then centrifuged for 10 minutes at 10,000 rpm. When all of the nuclei were collected in a pellet, the supernatant was discarded. The pellet was then resuspended in 600 μL of PBS plus 0.5% Triton X-100. The number of nuclei was estimated by staining with propidium iodide (PI), a fluorescent molecule that stoichiometrically binds to DNA by intercalating between the bases with no sequence preference. Aliquots of 25 μL were resuspended in 200 μL of a 0.2 M solution of boric acid, pH 9.0, and heated for 1 hour at 75°C for epitope retrieval. After washing in PBS, the nuclei were incubated for 24 hours at 4°C with primary mouse monoclonal anti-BrdU antibody (1:100, Abcam, Ab12219, Cambridge, UK) and subsequently incubated with FITC-conjugated goat anti-mouse IgG secondary antibody (1:100 in PBS; Vector Labs, FI-2001). The remainder of the cell suspension was diluted to 500 uL and sent for flow cytometry assay using Beckman FC 500 System with CXP Instrument Manager v.8.05 (Brea, CA, USA). PI cells were first gated on a histogram; the expressing cells were visualized on a forward/side scatter plot. PI cells were “back-gated” on the forward/side scatter plot to eliminate debris prior to analysis; this also eliminated auto fluorescence of the sample. Gates were always set using dissociates with cell aliquots which lack of the first antibody, but which were incubated with secondary antibody and processed alongside the experimental procedure. PI-labeled cells in a fixed volume were gated, and the number of cells showing BrdU+ signal was analyzed.

### Balance beam

The effect of Allo on motor coordination and balance in rats was measured by the use of a balance beam paradigm [28, 29]. The apparatus is a 60 cm long wooden dowel rod (diameter = 5 cm) attached to a 25 cm x 35 cm smooth-surfaced escape platform, leveled and suspended by a stable support column 60 cm above the floor, protected by a thick soft foam-padded surface to ensure safety. The beam height was sufficient to deter the rat from jumping to the floor. On each experimental day, the rats were weighed and habituated to the balance beam apparatus prior to treatment injection as a baseline control test. At baseline, all rats were able to walk along the rod to reach the platform within the time limit of 120 sec. After treatment, rats were then re-tested for their ability to walk across the rod at 5 min intervals until recovery. For each trial, a rat was positioned with their head near the center point of the rod facing the platform and was scored for their ability to walk across the remaining 30 cm on top of the rod, to reach the escape platform within a time limit of 120 sec. Measured from the hindlegs or base of their tail, the test required the rat to walk approximately 60 cm to fully escape from the rod onto the platform. Each rat was compared to its own baseline to determine its sedation level over time. The task was designed to be sufficiently simple enough that at baseline each rat could reach the platform every time and that recovery from acute sedation could be accurately assessed at timed intervals. The balance beam was thoroughly cleaned in between trials. Doses were based on individual body weight. A washout period of 3 days up to 1 week between experiments was allowed to avoid acute tolerance. Rats were individually tested in a room with dimmed lighting, white noise background, and ranked by two observers. A rank score was determined using the following 5-point scale: 0-fall, 1-clasp, 2-all paws on top, 3-takes steps, 4-reach the platform [28]. Area bound by the sedation curve (AUC_sed_) was calculated using the trapezoidal method and expressed as behavioral score (a rank score between 4 and 0 described above) multiplied by minutes post-treatment.

### Statistical analysis

Statistical significance for group comparison was performed by a planned one-way ANOVA or Student’s *t*-test followed by a Newman-Keuls post-hoc analysis. The non-parametric Wilcoxon signed-rank test or Mann-Whitney U-test analyzed ranked behavioral scores. The difference between groups was considered significant when the P value was < 0.05.

## Results

### Pharmacokinetics of allopregnanolone in two species

**Fig 1:**
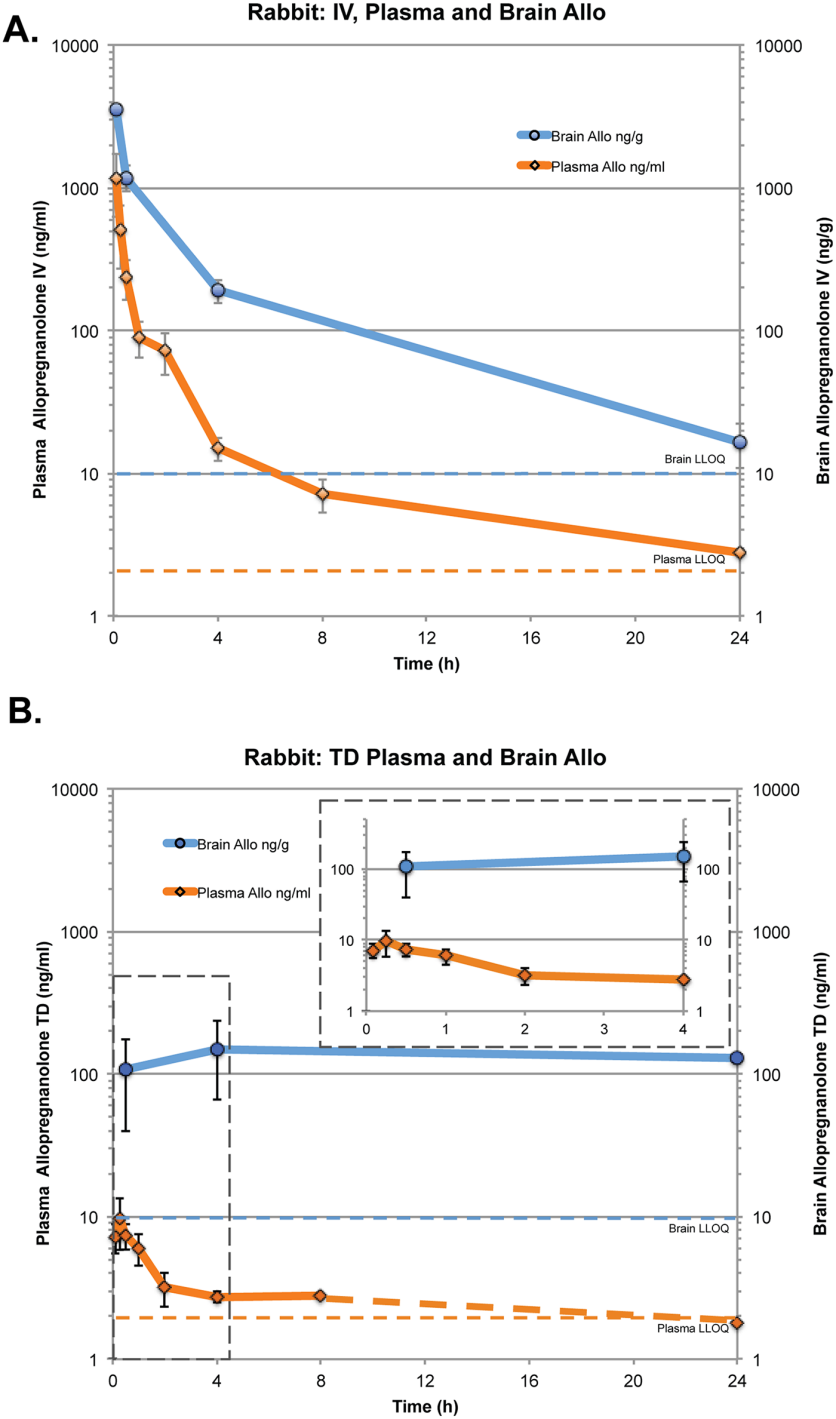
Rabbit plasma and brain concentration-time profiles of allopregnanolone following intravenous (IV) bolus or transdermal (TD) administration. A. Dosing at 3 mg/kg IV bolus using 1.5 mg/ml Allo solution in 20% HBCD in nine female New Zealand rabbits (*n* = 1–9 per time point) and B. Dosing 5 mg/kg TD using 20 mg/ml Allo solution in DMSO administered to nine adult female New Zealand rabbits (*n* = 1–9 per time point). Data points represented as mean ± SD. LLOQ: Lower Limit of Quantification.

**Fig 2:**
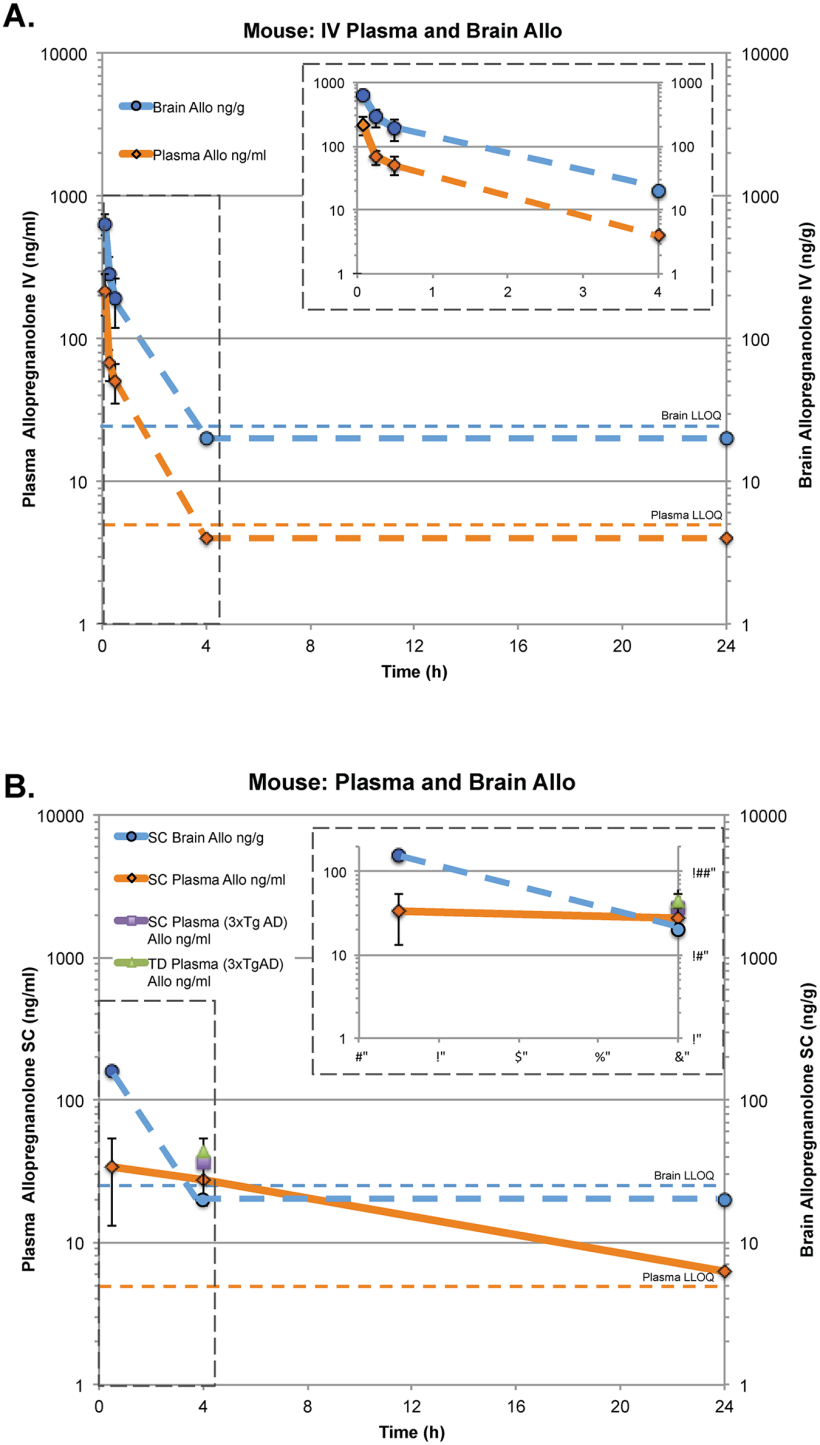
Mouse plasma and brain concentration-time profiles of allopregnanolone following intravenous (IV), subcutaneous (SC) and transdermal (TD) administration. A. Dosing at 1.5 mg/kg IV using Allo solution in 6% HBCD vehicle in male non-transgenic mice (15-months-old, *n* = 5–6). B. Dosing at 10 mg/kg SC using Allo suspension in 5% EtOH/PBS vehicle in male non-transgenic mice (15-months-old, *n* = 1–3), at 10 mg/kg SC using Allo suspension in 5% EtOH/PBS vehicle in male 3xTgAD mice (5-months-old, *n* = 6) and 50 mg/kg TD using Allo solution 45% DMSO, 30% EtOH, 2.5% Klucel MF, 19.2% PEG-300 (5-months-old, *n* = 8). Data points represented as mean ± SD. LLOQ: Lower Limit of Quantification.

PK parameters of Allo were determined in plasma and brain of female rabbits after intravenous (IV) administration and male mice after IV and subcutaneous (SC) administration. Across two species and routes of administration, the PK profiles were consistent (Figs 1 and 2; Table 1). Plasma and brain samples of both species tested showed equal T_max_ values (0.08 h). Brain Allo levels reached as high as C_max_ 3550 ng/ml at 5 min post-IV bolus measured post-mortem in one of the 9 rabbits that received IV Allo 3 mg/kg. At 30 min post-dose, whole brain Allo in 3 rabbits was 1182±241 ng/ml. The IV Allo elimination half-life (T_1/2_) in plasma was 3.3 h and 4.0 h and similar in brain, 3.7 h and 4.0 h, in rabbits and mice respectively. The elimination rate constants were also similar between species and tissues, ranging from 0.209–0.189 hr^-1^ for rabbits and from 0.174–0.224 hr^-1^ for mice.

**Table 1:** Pharmacokinetic parameters for allopregnanolone in both rabbit and mouse.

**PLASMA**												
**Species**	**Dose Route**	**Dose Level (mg/kg)**	**C**_**max**_ **(ng/ml)**	**T**_**max**_ **(hr)**	**AUC**_**last**_ (hr*ng/ml)	**AUC**_**inf**_ (hr*ng/ml)	**t**_**1/2**, **1**_ **(hr)**	**t**_**1/2**, **z**_ **(hr)**	***k***_***elimin***_ **(hr** ^**-1**^ **)**	**V**	**Cl**	**F**
										**L/kg**	**ml/hr/kg**	**%**
Rabbit	IV	3	1176	0.08	611.9	625.1	0.26	3.3	0.209	23.0	4799.2	N/A
Mouse	IV	1.5	214.6	0.08	149.9	155.6	0.21	4	0.174	55.4	9640.1	N/A
Mouse	SC	10	33.5	0.5	445.9	533.1	12.46	9.6	0.072	132.9	9566.7	51
Rabbit	TD	5	9.6	0.25	58.9	71.2	2.14	8.5	0.082	58.2	4775.3	6.8
**BRAIN**												
**Species**	**Dose Route**	**Dose Level (mg/kg)**	**C**_**max**_ **(ng/g)**	**T**_**max**_ **(hr)**	**AUC**_**last**_ (hr*ng/g)	**AUC**_**inf**_ (hr*ng/g)	**t**_**1/2, 1**_ **(hr)**	**t**_**1/2, z**_ **(hr)**	***k***_***elimin***_ **(hr** ^**-1**^ **)**	**V**	**Cl**	**F**
										**L/kg**	**ml/hr/kg**	**%**
Rabbit	IV	3	3550	0.08	5464.7	5554.5	0.27	3.7	0.189	25.4	4799.2	N/A
Mouse	IV	1.5	639.2	0.08	496.5	501	0.25	4	0.224	44.7	10006.7	N/A
Mouse	SC	10	159	0.5	300	307	0.48	4.9	0.142	80.5	11437.5	9
Rabbit	TD	5	151.0	4.0	3261.5	**Insufficient data to calculate**						35.8

LC-MS/MS measurements of allopregnanolone in plasma (top panel) or brain (bottom panel) after a single bolus dose (mg/kg body weight) by intravenous (IV), subcutaneous (SC), or transdermal (TD) administration.

### Bridging subcutaneous to intravenous route of administration

PK analysis was conducted in 15-month-old wildtype male mice, administered Allo 10 mg/kg SC or 1.5 mg/kg IV in a 24 h time course (Fig 1B; Table 1). At 30 min following SC administration, the Allo plasma concentration was 34±20.4 ng/ml (107 nmol/L), which is comparable to the plasma concentration 51 ng/ml (160 nmol/L) 30min after IV administration. Brain cortex concentrations were also comparable at 159 ng/g, 30min after SC administration and 192 ng/g, 30min after IV administration. The mean absolute bioavailability of SC administered Allo was 44% in plasma with a T_1/2_ of 9.6 h. For the SC time course conducted in aged wildtype mice, plasma Allo concentrations were 27.6±8.7 ng/ml 4 h after treatment, which is comparable to 5-month-old 3xTgAD male mice Allo 36.2±18.2 ng/ml treated with the same SC formulation (10 mg/kg; 5%EtOH/PBS) (Fig 2B). By comparison, at 4 h the IV dosing group plasma and brain levels were below the quantifiable limits (Fig 2A).

### Transdermal delivery

In rabbits, transdermal (TD; topical) Allo 5 mg/kg dose reached peak plasma C_max_ of 9.6 ng/ml (30 nmol/L) at 15 min, a much lower peak concentration compared to IV Allo 3 mg/kg dose (Fig 1A and 1B; Table 1). Systemic exposure to Allo was 6.8% following TD administration (Fig 1B; Table 1), compared to the 100% for the IV dose route (Fig 1A). The concentration of TD administered Allo in the brain was higher than IV administered plasma Allo level after approximately 8 h (Fig 1A and 1B). Interestingly, Allo levels above 100 ng/g were maintained in the brain for the full 24 h study time course, resulting in brain exposure (AUC) in the TD group that was 36% of the IV group. In 5-month-old 3xTgAD male mice, plasma levels were 35.5±19.8 ng/ml 4 h after TD dose of 50 mg/kg using a topical gel formulation (Fig 2B).

### Effect of subcutaneous Allo on markers of neurogenesis in 3xTgAD mice

**Fig 3:**
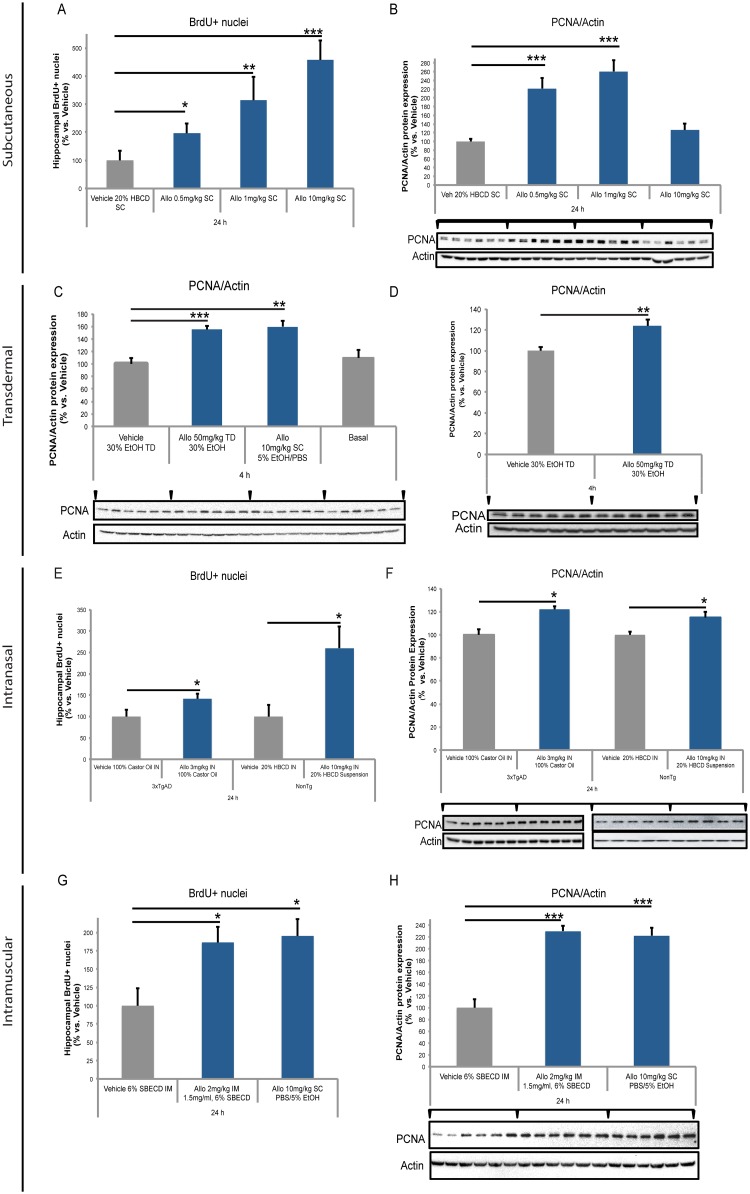
Subcutaneous Allo increased BrdU incorporation and PCNA protein expression in male mouse AD model. A. In 5-month-old 3xTgAD mouse hippocampus, BrdU+ nuclei increased significantly in 24 h after a single subcutaneous (SC) dose of Allo 0.5, 1, or 10 mg/kg. B. In 5-month-old 3xTgAD mouse hippocampus, protein expression of ~29 kDa PCNA increased significantly at 24 h after SC Allo 0.5 and 1 mg/kg doses whereas 10 mg/kg dose trended towards increase but did not reach significance. Transdermal and subcutaneous Allo increased PCNA protein expression in male mouse AD model. C. In 5-month-old 3xTgAD mouse hippocampus, protein expression of PCNA increased significantly at 4 h after transdermal (TD) Allo 50 mg/kg and SC Allo 10 mg/kg doses. D. In 15-month-old nonTg mouse hippocampus, protein expression of PCNA increased significantly at 4 h after TD Allo 50 mg/kg dose. E. In 5-month-old 3xTAD and 15-month-old nonTg mouse hippocampus, BrdU+ nuclei increased significantly in 24 h after intranasal (IN) dose of Allo 3 mg/kg 100% Castor Oil and Allo 10 mg/kg 20% HBCD suspension doses. F. In 5-month-old 3xTgAD and 15-month-old nonTg mouse hippocampus, protein expression of PCNA increased significantly at 24 h after IN Allo 3 mg/kg 100% Castor Oil and Allo 10 mg/kg 20% HBCD suspension doses. Intramuscular Allo-induced increase in cell cycle marker in male mouse AD model. G. In 5-month-old 3xTgAD mouse hippocampus, BrdU+ nuclei increased significantly in 24 h after a single intramuscular (IM) dose of Allo 2 mg/kg and SC Allo 10 mg/kg dose. H. In 5-month-old 3xTgAD mouse hippocampus, protein expression of PCNA increased significantly at 24 h post-IM Allo 2 mg/kg dose and SC Allo 10 mg/kg dose. Intravenous Allo-induced increase in cell cycle and neurodifferentiation markers in male mouse AD model. I. In 5-month-old 3xTgAD mouse hippocampus, protein expression of 30 kDa cyclinD2 increased significantly at 4 h post-intravenous (IV) Allo 0.1 and 0.5 mg/kg dose whereas 1 mg/kg dose trended towards increase but did not reach significance. J. In 5-month-old 3xTgAD mouse hippocampus, protein expression of PCNA increased significantly at 4 h after IV Allo 0.5 mg/kg dose whereas 0.1 and 1 mg/kg dose did not reach significance. K. In 5-month-old 3xTgAD mouse hippocampus, protein expression of ~40 kDa doublecortin (DCX) increased significantly at 4 h after IV Allo 0.5 and 1 mg/kg doses. L. In 5-month-old 3xTgAD mouse hippocampus, protein expression of 49 kDa NeuroD increased significantly at 4 h after IV Allo 0.5 mg/kg, whereas 0.1 and 1 mg doses did not reach significance. Intravenous Allo-induced rapid transient increase in CREB phosphorylation in male mouse aging model. M. In 15-month-old nonTg mouse hippocampus, protein expression of 43 kDa serine 133 phosphorylated CREB (pCREB) increased significantly 5 min after IV Allo 1.5 mg/kg dose. N. In 15-month-old nonTg mouse hippocampus, protein expression of 49 kDa NeuroD1 (NeuroD) increased significantly at 4 h then decreased at 24 h after intravenous Allo 1.5 mg/kg dose. * p<0.05, ** p<0.01, *** p<0.001, **** p<0.0001, *n* = 4–6, bars represent mean value ± SEM.

To develop the optimal therapeutic dose of Allo for AD by the SC route of administration, we investigated dose-ranges with cyclodextrin-solubilized formulations (Fig 3; Table 2). While a suspension formulation would be acceptable by SC route, only soluble particle-free solutions are clinically acceptable by IV route [30]. To bridge our previous studies with SC soluble 20% 2-hydroxyl-beta-cyclodextrin (HBCD) [12] to IV route solubilized with cyclodextrins (HBCD or sulfobutyl-ether-beta-cyclodextrin (SBECD)) in a model relevant to human disease, we conducted analyses in both aged wildtype and young 3xTgAD mouse models that were previously confirmed to respond to SC Allo administration (Wang et al 2010; Chen et al. 2011, Singh et al. 2011). To further evaluate the neurogenic efficacy of Allo in mice, a single SC bolus treatment paradigm with thymidine analog BrdU was injected 1h following Allo administration. Results from flow cytometry analysis indicated that SC Allo treatment in 5-month-old 3xTgAD mouse hippocampus significantly increased S-phase DNA synthesis labeled by BrdU+ nuclei (Fig 3A; Table 2). Relative to vehicle control, SC Allo increased proliferation in a dose dependent manner with 0.5 mg/kg inducing a 95 ± 35% increase (p<0.05), 1 mg/kg inducing a 215 ± 83% increase (p<0.01), and 10 mg/kg increasing 360 ± 68% (p<0.001). At the same 24 h time point, Allo SC 0.5 mg/kg increased proliferating cell nuclear antigen (PCNA) expression relative to vehicle by 122 ± 24% (p<0.001) and 1 mg/kg increased expression by 160 ± 25% (p<0.001) in the 3xTgAD mouse hippocampus (Fig 3B). At 24 h, PCNA expression did not reach statistical significance with SC Allo 10 mg/kg, although there was a trend towards increased expression. PCNA is a cofactor of DNA polymerases and is an S-phase cell cycle marker that we have previously shown to be a surrogate indicator of Allo-induced neurogenesis [10].

**Table 2:** Summary of mouse efficacy data and statistical comparisons.

Route of Administration	Mouse Model	Allo Dose	Timepoint	Biomarker	Allo-induced % Change Relative to Vehicle	P-value
Study 1: Soluble SC dose response, 24h (Fig 3A and 3B)						
SC	3xTgAD, 5mo	0.5mg/kg	24h	BrdU	95 ± 35% incr.	<0.05
	3xTgAD, 5mo	1mg/kg	24h	BrdU	215 ± 83% incr.	<0.01
	3xTgAD, 5mo	10mg/kg	24h	BrdU	360 ± 68% incr.	<0.001
	3xTgAD, 5mo	0.5mg/kg	24h	PCNA	122 ± 24% incr.	<0.001
	3xTgAD, 5mo	1mg/kg	24h	PCNA	160 ± 25% incr.	<0.001
Study 2A: TD vs. susp. SC single dose, 4h (Fig 3C)						
TD	3xTgAD, 5mo	50mg/kg	4h	PCNA	55 ± 6% incr.	<0.001
SC	3xTgAD, 5mo	10mg/kg	4h	PCNA	60 ± 10% incr.	<0.01
Study 2B: TD single dose, 4h (Fig 3D)						
TD	WT, 15mo	50mg/kg	4h	PCNA	24 ± 6% incr.	<0.01
Study 3: Soluble IN vs. susp. IN single dose, 24h (Fig 3E and 3F)						
IN	3xTgAD, 5mo	3mg/kg	24h	BrdU	42 ± 12% incr.	<0.05
	WT, 15mo	10mg/kg	24h	BrdU	160 ± 51% incr.	<0.05
	3xTgAD, 5mo	3mg/kg	24h	PCNA	22 ± 3% incr.	<0.001
	WT, 15mo	10mg/kg	24h	PCNA	15 ± 5% incr.	<0.05
Study 4: IM vs. susp. SC single dose, 24h (Fig 3G and 3H)						
IM	3xTgAD, 5mo	2mg/kg	24h	BrdU	86 ± 22% incr.	<0.05
SC	3xTgAD, 5mo	10mg/kg	24h	BrdU	95 ± 23% incr.	<0.05
IM	3xTgAD, 5mo	2mg/kg	24h	PCNA	130 ± 10% incr.	<0.001
SC	3xTgAD, 5mo	10mg/kg	24h	PCNA	123 ± 14% incr.	<0.001
Study 5: IV dose response, 4h (Fig 3I, 3J, 3K and 3L)						
IV	3xTgAD, 5mo	0.1mg/kg	4h	CyclinD2	32 ± 11% incr.	<0.05
	3xTgAD, 5mo	0.5mg/kg	4h	CyclinD2	40 ± 8% incr.	<0.01
	3xTgAD, 5mo	0.5mg/kg	4h	PCNA	25 ± 3% incr.	<0.05
	3xTgAD, 5mo	0.5mg/kg	4h	DCX	60 ± 17% incr.	<0.05
	3xTgAD, 5mo	1mg/kg	4h	DCX	50 ± 23% incr.	<0.05
	3xTgAD, 5mo	0.5mg/kg	4h	NeuroD	18 ± 4% incr.	<0.05
Study 6: IV time course (Fig 3M and 3N)						
IV	WT, 15mo	1.5mg/kg	5min	pCREB	90 ± 9% incr.	<0.001
	WT, 15mo	1.5mg/kg	15min	pCREB	130 ± 11% (decr. relative to peak pCREB)	<0.0001
	WT, 15mo	1.5mg/kg	15min	pCREB	40 ± 11% (decr. relative to basal pCREB)	<0.05
	WT, 15mo	1.5mg/kg	4h	NeuroD	43 ± 10% incr.	<0.01
	WT, 15mo	1.5mg/kg	24h	NeuroD	34 ± 7% incr.	<0.01

Summary of data presented in Fig 3. Study 1 Allo subcutaneous (SC) dose response demonstrated increased hippocampal BrdU-labeled nuclei and PCNA protein levels in early pathology-stage 3xTgAD mice. Study 2A demonstrated comparable hippocampal PCNA increases 4 h after transdermal (TD) and our previously reported (Wang et al. 2010) suspension SC Allo. Study 2B extended TD Allo-induced hippocampal PCNA increase at 4 h to 15-month-old wildtype (nontransgenic background strain) mice. Study 3 demonstrated that both 3 mg/kg and 10 mg/kg intranasal (IN) Allo increased hippocampal BrdU-labeled nuclei and PCNA protein levels. Study 4 demonstrated that intravenous (IV) sub-sedative doses ranging from 0.1 mg/kg to 1 mg/kg increased cell proliferation and differentiation markers cyclinD2, PCNA, doublecortin, and NeuroD with 0.5 mg/kg dose efficacious across all markers in young adult 3xTgAD mice. Study 5 was conducted with the same wildtype 15-month-old mice that were used to conduct the PK study (see Fig 2B; Table 1). Allo 1.5 mg/kg IV induced a rapid rise in CREB phosphorylation that followed the PK Allo peak and rapid clearance. NeuroD, an important transcription factor in cell maturation to neurons was expressed within 4 h after a single bolus dose of Allo.

### Effect of transdermal and intranasal Allo on neurogenesis markers in both mouse Alzheimer’s disease and aging models

In 5-month-old 3xTgAD mouse hippocampus, Allo TD 50 mg/kg increased PCNA expression relative to vehicle by 55 ± 6% (p<0.001) at 4 h after topical gel application, had a comparable PCNA increase of 60 ± 10% (p<0.01) relative to vehicle compared to suspension SC Allo 10 mg/kg dose (Fig 3C). Basal or untreated mice had the same expression of PCNA as the TD vehicle group. In 15-month-old nonTg mouse hippocampus, protein expression of PCNA increased 24 ± 6% (p<0.01) at 4 h (Fig 3D) after TD Allo 50 mg/kg dose, comparable to the expression response observed in young 3xTgAD mice (Fig 3C). In addition to TD as a topical route, intranasal (IN) was tested in both 5-month-old 3xTgAD and 15-month-old nonTg mouse hippocampus. BrdU+ nuclei increased significantly 42 ± 12% (p<0.05) after IN dose of Allo 3 mg/kg 100% castor oil and by 160 ± 51% (p<0.05) after IN Allo 10 mg/kg 20% HBCD suspension, measured 24 h after dosing (Fig 3E). Hippocampal protein expression of PCNA increased 22 ± 3% (p<0.001) at 24 h after IN Allo 3 mg/kg 100% castor oil and 15 ± 5% (p<0.05) after Allo 10 mg/kg 20% HBCD suspension doses (Fig 3F).

### Effect of intramuscular Allo on markers of neurogenesis in 3xTgAD mice

We investigated neurogenic efficacy of Allo administered by IM route in 6% SBECD (Cyclolab, Ltd) molar ratio 5.89. Results from flow cytometry analysis indicated that IM Allo treatment in 5-month-old 3xTgAD mouse hippocampus significantly increased BrdU+ labeled nuclei (Fig 3G; Table 2). Relative to vehicle control, IM Allo increased proliferation 2 mg/kg inducing an 86 ± 22% increase (p<0.05) and comparable to SC suspension 10 mg/kg 95 ± 23% increase (p<0.05) within 24 h. At the same time point, Allo IM 2 mg/kg increased PCNA expression relative to vehicle by 130 ± 10% (p<0.001) and comparable to SC suspension 10 mg/kg by 123 ± 14% (p<0.001) in the 3xTgAD mouse hippocampus (Fig 3H). Neither IM 2 mg/kg nor SC suspension 10 mg/kg were sedative.

### Effect of intravenous Allo on markers of neurogenesis and neuronal differentiation in 3xTgAD mice

To bridge from SC to IV route of administration, Allo dose-response studies in 5-month-old 3xTgAD mice after SC and IV administration, and in 15-month old wildtype mice after IV administration were conducted. Neurogenic efficacy was measured by hippocampus protein expression of cell cycle proliferation and differentiation markers. Our previous studies indicated dose-dependent efficacy of Allo on neurogenesis [10, 11].

Neurogenic efficacy of Allo was assessed in the hippocampus of 5-month-old 3xTgAD mice by Western blot analyses of several rapidly induced cell cycle proteins. Expression of 30 kDa cyclinD2 increased 32 ± 11% (p<0.05) and 40 ± 8% (p<0.01) at 4 h with IV Allo 0.1 and 0.5 mg/kg doses respectively (Fig 3I). CyclinD2 is the only D-type cyclin expressed in dividing cells derived from neuronal precursors present in the adult hippocampus [31]. Protein expression of PCNA increased 25 ± 3% (p<0.05) at 4 h after an IV Allo 0.5 mg/kg dose whereas 0.1 and 1mg/kg doses did not reach significance (Fig 3J).

DCX is a microtubule-associated protein that is expressed specifically in migrating neuronal precursors of the developing CNS [32] and correlates with the level of cellular proliferation in the dentate gyrus [33] [34]. Protein expression of DCX increased at 4 h after IV Allo 0.5 and 1mg/kg doses by 60 ± 17% and 50 ± 23% respectively (Fig 3K).

To assess impact of Allo on phenotypic differentiation to neurons, protein expression of NeuroD, a transcription factor required for differentiation of immature hippocampal granule cells to neurons was determined [35]. NeuroD increased by 18 ± 4% after IV Allo 0.5 mg/kg, whereas 0.1 and 1mg doses did not reach statistical significance (Fig 3L).

Collectively, these data indicate that 0.5 mg/kg IV Allo in a cyclodextrin-based formulation was the optimal sub-sedative dose for inducing protein expression of multiple neuroregenerative efficacy indicators in the early-Alzheimer’s disease pathology stage of 3xTgAD mice.

### Rapid effects of intravenous Allo on phospho-CREB and NeuroD expression in aged wildtype mice

Previously we established that 15-month-old wildtype mice had a significantly reduced basal level of BrdU+ cells and learning performance comparable to that of 6-month-old 3xTgAD mice [13]. Allo restored regenerative capacity of 15-month wildtype mice to neurogenic levels of 6-month wildtype levels [13]. To determine Allo neurogenic efficacy, we performed Western blot analyses and confirmed that hippocampal CREB activation was consistent with the Allo PK profile (Fig 2A) in the same 15-month-old wildtype male mice. IV Allo 1.5 mg/kg induced a rapid transient increase in phosphorylated CREB at 5 min (C_max_ brain), 90 ± 9% (p<0.001) relative to basal (Fig 3M). Phospho-CREB expression followed the same rapid peak and rapid elimination of Allo from brain and blood (Fig 2A). By 15 min, phospho-CREB was significantly decreased relative to peak (p<0.0001) and decreased 40 ± 11% (p<0.05) below basal. Collectively, these data indicate that Allo induced a transient activation of phospho-CREB followed by deactivation.

To determine whether induction of phospho-CREB resulted in downstream indicators of neurogenesis we investigated expression of a marker of neuronal differentiation, NeuroD. At 4 h post-IV infusion, Allo induced a significant increase (43 ± 10%; p<0.01) in NeuroD expression. As with induction of phospho-CREB, expression of NeuroD was transient with a significant decline (34 ± 7%; p<0.01) at 24 h relative to vehicle (Fig 3N).

### Effects of Allo:Cyclodextrin molar ratio on sedation in rats

**Fig 4:**
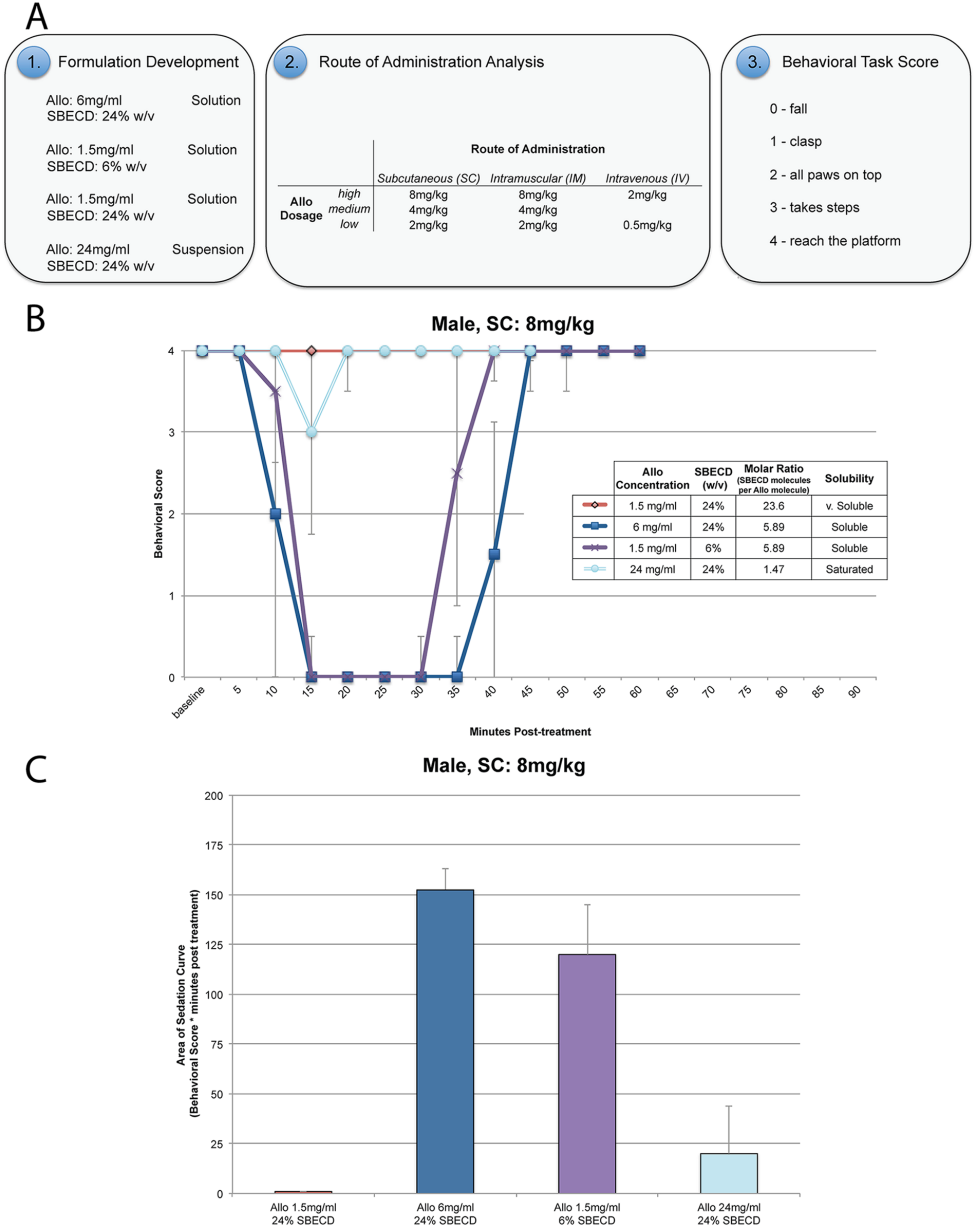
Formulation development, route of administration, and rat behavioral task study components. Study design. A. Four different formulations were administered by subcutaneous (SC) route to male rats at the maximally tolerated dose (MTD) for sedation, determined to be 8 mg/kg for the leading formulation Allo 6 mg/ml, 24% sulfobutyl-ether-β-cyclodextrin sodium (SBECD). Optimal release rate formulations for SC were then tested via dose-response for SC, intramuscular (IM), and intravenous (IV) routes using the rat balance beam behavioral task to score motor incoordination due to Allo-induced sedation, a biomarker of Allo target engagement and tolerability. Sedation score as a biomarker of Allo target engagement. B. The sedative component of GABA_A_ receptor activation in the brain was used as a biomarker outcome of Allo delivery and tolerability. Male rats (Age 7 months at study initiation) were dosed by SC route of administration with multiple formulations, to determine effect on sedation. Doses were administered at Allo 8 mg/kg SC to compare sedation responses at 5 min intervals. The formulations were of either Allo solutions or suspensions (in 0.9% sodium chloride with SBECD) designed to determine the Allo delivery rate as it relates to SBECD:Allo molar ratio. Balance Beam Behavioral Scoring System: 4 = reach platform; 3 = takes steps; 2 = all paws on top; 1 = clasp; 0 = fall. *n* = 4, interval points represent mean value ± SEM. Area bound by sedation curve as an indicator of rapid Allo target engagement. C. The area bound by the sedation curve (AUC_sed_; area above the curve as graphically represented) was calculated to determine the sedative component of GABA_A_ receptor activation in the brain as an indicator of Allo delivery. Male rats (Age 7 months at study initiation) were administered Allo 8 mg/kg SC bolus to compare delivery of multiple formulations of either Allo solutions or suspensions (in 0.9% sodium chloride with SBECD) designed to determine the Allo delivery rate as it relates to SBECD:Allo molar ratio. Area bound by sedation curve was calculated and expressed as behavioral score (a score between 4 and 0) multiplied by minutes post-treatment. *n* = 4, bars represent mean value ± SEM.

To establish the optimal formulation, the molar ratio of Allo to SBECD, to move forward in translational analyses, we analyzed the effect of the molar ratio on brain bioavailability as assessed through motor coordination and sedation. To determine the impact of SBECD:Allo molar ratio, a series of formulations in decreasing molar ratio was investigated in male rats (*n* = 4 per formulation). Allo was administered SC at a constant Allo dose of 8 mg/kg bolus injection in varying formulations. The 8 mg/kg Allo dose was used for these analyses to hold dose constant while varying molar ratio of the formulation. This dose was arrived at based on preliminary analyses that showed 8 mg/kg of Allo induced maximal sedation in 6 mg/ml Allo in 24% SBECD solution, an SBECD:Allo molar ratio of 5.89. All molar ratios were compared relative to the 5.89 formulation. Functional outcomes were scored using an ordinal behavioral task, where 0 indicated muscle flaccidity and loss of balance while a score of 4 indicated that the rat was able to complete the task with no discernible impairment in performance (Fig 4A).

Male rats dosed with 1.5 mg/ml Allo in 24% SBECD solution, an SBECD:Allo molar ratio of 23.56 displayed no sedation with balance beam performance comparable to pre-treatment baseline (Fig 4B and 4C; Table 3A).

**Table 3:** Summary of rat sedation data and statistical comparisons.

**Study 1: Formulation**									
**Route of Administration**	**Sex**	**Dose**	**Endpoint**	**Comparison**	**Mean ± SEM**	**Mean ± SEM**	**Median (Interquartile Range)**	**Median (Interquartile Range)**	**p-value**
SC	M	8mg/kg	Deepest sedation score	1.5mg/ml 6% (Molar Ratio 6) vs 1.5mg/ml 24% (Molar Ratio 24)	4 ± 0	0 ± 0	4(0)	0(0)	<0.0001
	M	8mg/kg	Deepest sedation score	6mg/ml 24% (Molar Ratio 6) vs 1.5mg/ml 24% (Molar Ratio 24)	4 ± 0	0 ± 0	4(0)	0(0)	<0.0001
**Study 2: Route of Administration**									
**Route of Administration**	**Sex**	**Dose**	**Endpoint**	**Mean ± SEM**	**Median (Interquartile Range)**	**Comparison**	**Mean ± SEM**	**Median (Interquartile Range)**	**p-value**
SC	M&F	4mg/kg	Deepest sedation score	2.75 ± 0.9	4(0.25)	8mg/kg (M&F)	0 ± 0	0(0)	0.0313
SC	M&F	4mg/kg	Duration of deepest sedation	13.75 ± 4.8	0(1.25)	8mg/kg (M&F)	31.25 ± 2.4	25(15)	0.0156
SC	F	8mg/kg	AUC_sed_	78.75 ± 26.9	97.5(37.5)	M	146.88 ± 9.8	132.5(46.1)	0.0571
SC	F	8mg/kg	Duration of deepest sedation	12.5 ± 6.0	12.5(17.5)	M	31.25 ± 2.4	32.5(6.25)	0.0571
IM	M&F	2mg/kg	Deepest sedation score	3.625 ± 0.4	4(0.25)	4mg/kg (M&F)	1.5 ± 1.1	0(4)	0.0625
IM	M&F	2mg/kg	Duration of deepest sedation	1.25 ± 1.2	0(1.25)	4mg/kg (M&F)	12.5 ± 5.9	12.5(25)	0.0625
IM	F	4mg/kg	AUC_sed_	19.38 ± 19.38	0(19.4)	M	120.63 ± 5.8	118.75(15.6)	0.0286
IM	M	4mg/kg	Duration of deepest sedation	22.5 ± 2.5	25(2.5)	F	2.5 ± 2.5	0(2.5)	0.0286
IM	M&F	4mg/kg	Duration of deepest sedation	12.5 ± 5.8	12.5(25)	8mg/kg (M&F)	44.38 ± 8.1	45(20)	0.0078
IM	F	8mg/kg	AUC_sed_	153.75 ± 24.8	150(71.25)	M	245 ± 17.1	240(35)	0.0571
IM	F	8mg/kg	Duration of deepest sedation	31.25 ± 5.2	32.5(16.25)	M	57.5 ± 3.2	57.5(7.5)	0.0286
IV	M&F	0.5mg/kg	Deepest sedation score	3.75 ± 0.3	4(0.25)	2mg/kg (M&F)	0 ± 0	0(0)	0.0078
IV	M&F	0.5mg/kg	Duration of deepest sedation	12.5 ± 3.2	0(0.625)	2mg/kg (M&F)	10 ± 1.8	10(3.75)	0.0078
IV	M&F	0.5mg/kg	AUC_sed_	0.78 ± 0.52	0(0.625)	2mg/kg (M&F)	58.75 ± 4.2	55(15)	0.0078

**Fig 5:**
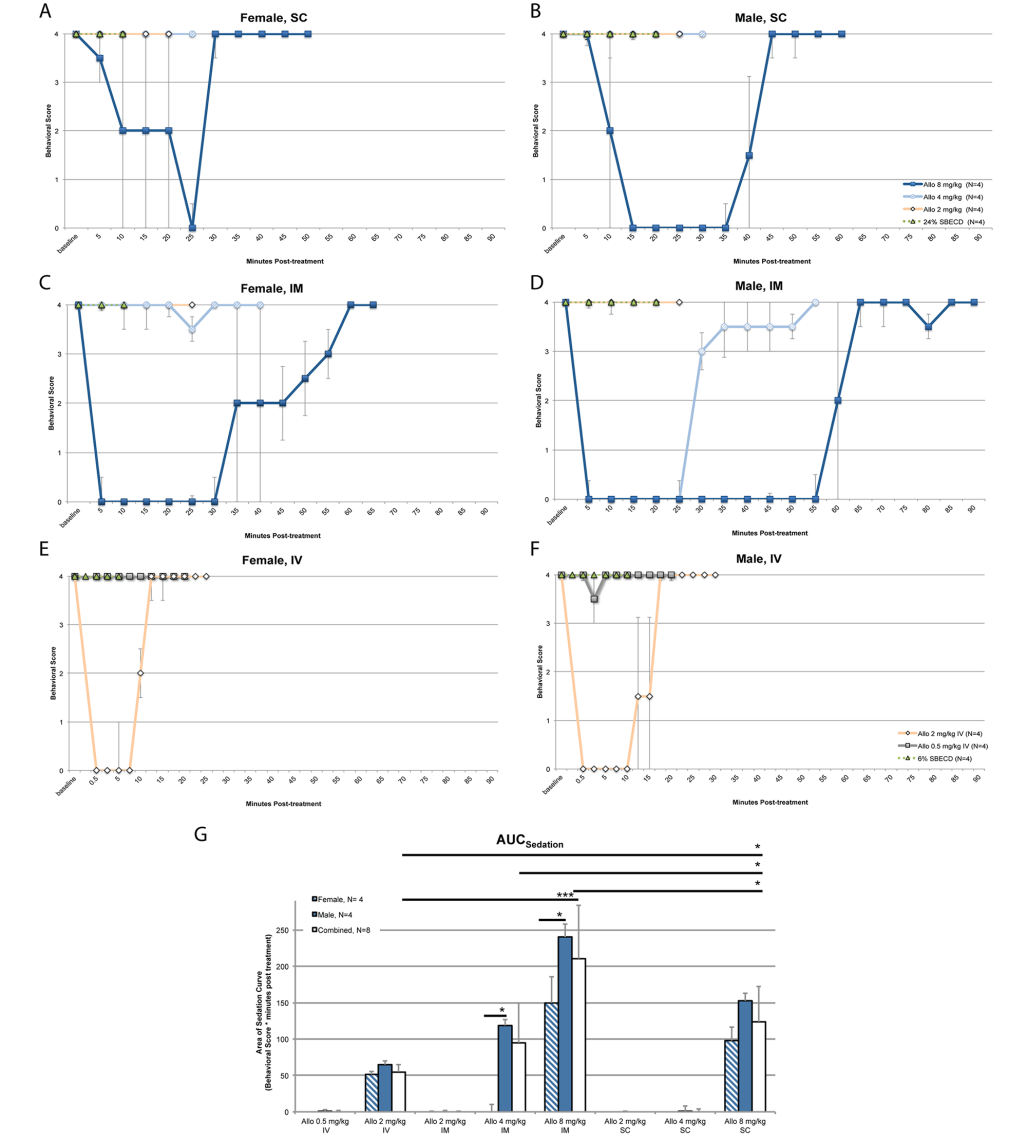
Allopregnanolone (Allo) dose-response comparison by subcutaneous (SC), intramuscular (IM), and intravenous (IV) routes of administration. Ovariectomized female rats (left panel) were compared to age-matched male rats (right panel) following SC (A. and B.), IM (C. and D.), or IV (E. and F.) bolus injection and challenged with a motor coordination behavioral task to assess level of sedation at timed intervals until recovery. SC/IM formulation: 6 mg/ml Allo solution in 0.9% sodium chloride with 24% sulfobutyl ether β-cyclodextrin sodium (SBECD). IV formulation: 1.5 mg/ml Allo solution in 0.9% sodium chloride with 6% SBECD. The volume of vehicle administered was equal to the volume administered with the highest dose, i.e. 8 mg/kg (SC/IM) or 2 mg/kg (IV). Balance Beam Behavioral Scoring System: 4 = reach platform; 3 = takes steps; 2 = all paws on top; 1 = clasp; 0 = fall. *n* = 4, interval points represent mean value ± SEM. Area bound by sedation curve as an indicator of rapid Allo target engagement. G. The area bound by the sedation curve (above the curve; Fig 5A–5F) (AUC_sed_) was calculated to determine the sedative component of GABA_A_ receptor activation in the brain as an indicator of Allo delivery. AUC_sed_ was calculated and expressed as behavioral score (a score between 4 and 0) multiplied by minutes post-treatment. Sex differences in the Allo-induced sedation response were determined. * p<0.05, ** p<0.01, *n* = 4, bars represent mean value ± SEM.

A. Four different formulations were administered by subcutaneous (SC) route to male rats at the maximally tolerated dose (MTD) for sedation, determined to be 8 mg/kg for the leading formulation Allo 6 mg/ml, 24% sulfobutyl-ether-β-cyclodextrin sodium (SBECD) (See Fig 4). B. Optimal release rate formulations determined via SC were then tested via dose-response for SC, intramuscular (IM), and intravenous (IV) routes using the rat balance beam behavioral task to score motor incoordination due to Allo-induced sedation, a biomarker of Allo target engagement and tolerability (See Fig 5). Units for comparison of mean ± SEM: Deepest sedation score (Balance Beam Behavioral Scoring System: 4 = reach platform; 3 = takes steps; 2 = all paws on top; 1 = clasp; 0 = fall); duration of deepest sedation (minutes post-treatment); Area bound by sedation curve (AUC_sed_; behavioral score * min post-treatment).

The reference formulation of 6 mg/ml Allo in 24% SBECD solution, an SBECD:Allo molar ratio of 5.89, displayed significant sedation and impaired motor coordination with complete motor impairment 15 min post-injection; median duration of maximal sedation was 32.5 min (interquartile range, 26–35 min) (Fig 4B). Total sedation was calculated for the area bound by the curve (above the curve) (AUC_sed_): AUC_sed_ median, 152.5 behavioral score * min post-treatment (interquartile range, 126–162 behavioral score * min post-treatment) (Fig 4C).

At 1.5 mg/ml Allo in 6% SBECD solution, an SBECD:Allo molar ratio of 5.89, displayed significant sedation and impaired motor coordination with complete motor impairment 20 min post-injection; median duration of maximal sedation was 20 min (interquartile range, 12.5–27.5 min) (Fig 4B). Total sedation was calculated: AUC_sed_ median, 120 behavioral score * min post-treatment (interquartile range, 70–142 behavioral score * min post-treatment) (Fig 4C).

At 24 mg/ml Allo in 24% SBECD suspension, an SBECD:Allo molar ratio of 1.47 displayed minor impairment (median, 2.5 min; interquartile range, 0–8.75 min), peaking at 15 min post-injection, with complete recovery by 30 min (Fig 4B). Total sedation was calculated: AUC_sed_ median, 20 behavioral score * min post-treatment (interquartile range, 0–62.5 behavioral score * min post-treatment) (Fig 4C).

Collectively, results of these analyses indicate that the SBECD:Allo formulation at a 5.89 molar ratio was optimal. This conclusion is based on the rapid induction of maximal sedation with greatest duration indicating optimal brain delivery relative to the other molar ratios.

### Effects of route of administration on sedation in rats

To investigate the impact of route of administration on sedation, we utilized the optimal SBECD:Allo 5.89 molar ratio formulation. Investigated routes of administration include SC, IM, and IV. Functional outcomes were scored using the same balance beam behavioral task used to determine the optimal formulation.

SC administration of 2 mg/kg or 4 mg/kg Allo to female (*n* = 4; Fig 5A and 5G) and male (*n* = 4; Fig 5B and 5G) rats did not induce sedation (behavioral score 4) whereas 8 mg/kg Allo induced significant sedation with median maximal behavioral score for female 0 (interquartile range, 0–3); duration of maximal sedation 12.5 min (interquartile range, 1.25–23.75 min); maximal behavioral score for male 0 (interquartile range, 0); duration 32.5 min (interquartile range, 26.25–35 min). Total sedation was calculated: females AUC_sed_ median, 97.5 behavioral score * min post-treatment (interquartile range, 23–115.5 behavioral score * min post-treatment) and males AUC_sed_, 152.5 behavioral score * min post-treatment (interquartile range, 126.5–162 behavioral score * min post-treatment) (Fig 5G).

IM administration of 2 mg/kg Allo to female (*n* = 4; Fig 5C and 5G) and male (*n* = 4; Fig 5D and 5G) rats did not induce appreciable sedation (median maximal behavioral score for female 4 (interquartile range, 3.25–4); maximal behavioral score for male 4 (interquartile range, 2.5–4)). IM 4 mg/kg Allo had sedative impact (maximal behavioral score for female 4 (interquartile range, 1–4); duration of maximal sedation 0 min (interquartile range, 0–7.5 min); maximal behavioral score for male 0 (interquartile range, 0); duration of maximal sedation 25 min (interquartile range, 17.5–25 min)). Total sedation was calculated: females AUC_sed_ median, 0 behavioral score * min post-treatment (interquartile range, 0–58 behavioral score * min post-treatment) and males AUC_sed_, 118.8 behavioral score * min post-treatment (interquartile range, 110.5–132.5 behavioral score * min post-treatment). Maximal sedative impact occurred with 8 mg/kg Allo IM administration (maximal behavioral score for female 0 (interquartile range, 0); duration of maximal sedation 32.5 min (interquartile range, 21.25–40 min); maximal behavioral score for male 0 (interquartile range, 0); duration of maximal sedation 57.5 min (interquartile range, 51.25–63.75 min)). Total sedation was calculated: females AUC_sed_ median, 150 behavioral score * min post-treatment (interquartile range, 109–202.5 behavioral score * min post-treatment) and males AUC_sed_, 240 behavioral score * min post-treatment (interquartile range, 215–280 behavioral score * min post-treatment) (Fig 5G).

IV administration of 0.5 mg/kg Allo to female (*n* = 4; Fig 5E and 5G) and male (*n* = 4; Fig 5F and 5G) rats did not induce sedation (median maximal behavioral score for female 4 (interquartile range, 4); maximal behavioral score for male 3.5 (interquartile range, 3–4)). At the highest dose tested, 2 mg/kg IV Allo, maximal sedative impact occurred (maximal behavioral score for female 0 (interquartile range, 0); duration of maximal sedation 7.5 min (interquartile range, 5.5–9.5 min); maximal behavioral score for male 0 (interquartile range, 0); duration of maximal sedation 12.5 min (interquartile range, 10–15 min)). Total sedation was calculated: females AUC_sed_ median, 50 behavioral score * min post-treatment (interquartile range, 45–55 behavioral score * min post-treatment) and males AUC_sed_, 70 behavioral score * min post-treatment (interquartile range, 57.5–75 behavioral score * min post-treatment) (Fig 5G).

Significant sex differences were apparent in duration of maximal sedation at IM 8 mg/kg Allo (female 32.5 min (range 21.25–40) vs males 57.5 min (range 51.25–63.75); p < 0.05) (Fig 5A–5F). There were also significant sex differences in total sedation area (AUC_sed_) at IM 4 mg/kg Allo (female 0 (range 0–58) vs males 118.8 (range 110.5–132.5) behavioral score * min post-treatment; p < 0.05). Although the following differences did not reach significance, the data suggests that with more animals a stronger difference may be detectable: duration of maximal sedation at SC 8 mg/kg Allo (female 12.5 min (range 1.25–23.75) vs males 32.5 min (range 26.25–35); p = 0.0571), and IV 2 mg/kg Allo (female 7.5 min (range 5.5–9.5) vs males 12.5 min (range 10–15); p = 0.0857); in AUC_sed_ SC 8 mg/kg Allo (female 97.5 (range 23–115.5) vs males 152.5 (range 126.5–162) behavioral score * min post-treatment; p = 0.0571; Fig 5A and 5B), IM 8 mg/kg Allo (female 150 (range 109–202.5) vs males 240 (range 215–280) behavioral score * min post-treatment; p = 0.0571), and IV 2 mg/kg Allo (female 50 (range 45–55) vs males 70 (range 57.5–75) behavioral score * min post-treatment; p = 0.0857) (Fig 5G).

Route of administration impacted the duration of sedation with 8 mg/kg SC resulting in shorter duration of maximal sedation relative to IM administration (p = 0.016, *n* = 8). Comparing IM to SC at equivalent doses, the level and extent of sedation was increased via the IM route, likely due to the high vascularization of quadricep muscle tissue allowing for more rapid and extensive systemic absorption. Within 30 sec of bolus administration, IV 2 mg/kg Allo induced maximal sedation. Results of route of administration analyses indicate that SBECD:Allo formulation at a 5.89 molar ratio administered via IM bolus was optimal. This conclusion is based on the rapid induction of maximal sedation with greatest duration indicating optimal brain delivery relative to the other routes.

Allo via IV and IM routes of administration displayed rapid onset of sedation with IV inducing the earliest onset IM inducing the greatest duration of sedation (Fig 5; Table 3B). In this regard, IV route of administration provides a method of determining the absolute bioavailable dose response. An IM bolus or IV slow infusion to mimic the PK profile of IM would provide clinical translatability with a longer time course for absorption and safe sedation profile.

### Estimation of the dose range from no observable adverse effect level (NOAEL) to maximally tolerated dose (MTD) for each route of administration in rats

Allo dose range finding via the sedation balance beam behavioral task was used to estimate the no observable adverse effect level (NOAEL) and maximally tolerated dose to sedation (MTD). The NOAEL was estimated to be Allo SC 4 mg/kg for female and SC 2 mg/kg for male rats (Fig 5A, 5B and 5G). Also from the behavioral data, we estimated an Allo NOAEL of IM 2 mg/kg for female and IM less than 2 mg/kg for male rats by bolus injection (Fig 5C, 5D and 5G).

For female rats treated with SC Allo, the MTD was estimated to be greater than 8 mg/kg. Female rats treated with Allo SC 8 mg/kg had a maximal median score of 0 (range 0–3) on the behavioral sedation scale indicating that full sedation was not reached in all animals and a slightly higher dose would be tolerated. For male rats treated with SC Allo, the MTD was estimated to be 8 mg/kg with a maximal behavioral score of 0 in all animals indicating loss of motor control. Similarly, the Allo IM MTD was estimated to be 8 mg/kg for female and less than 8 mg/kg for male rats with a maximal behavioral score of 0 in all animals.

To assess sedation following IV administration, the dose was adjusted to account for 100% bioavailability. Because Allo IV 1 mg/kg was found to be efficacious in mice and is equivalent to a 0.5 mg/kg dose in a rat (Table 4), 0.5 mg/kg was selected as the IV starting dose in rats. After administration of Allo IV 0.5 mg/kg, female rats showed no discernible signs of sedation and males showed very minimal sedation. From this we estimate the NOAEL to be Allo 0.5 mg/kg IV for female and slightly less than 0.5 mg/kg IV for male rats (Fig 5E–5G; Table 5). The MTD was estimated to be Allo IV 2 mg/kg for both female and male rats with a maximal behavioral score of 0 in all animals. The MTD of 2 mg/kg IV Allo reached maximal sedation levels in both male and female within 30 sec (Fig 5), briefly matching depth of sedation compared to SC or IM MTD with loss of righting reflex observed. After IV bolus injection, a rapid recovery from sedation was observed in rats which correlated with the Allo IV PK profile that was determined in rabbit and mouse (Fig 1; Table 1).

**Table 4:** Allopregnanolone equivalent doses in mouse, rat, rabbit, and human.

Allopregnanolone equivalent dose estimates						
Adult Species:	Mouse	Rat	Rabbit	Human	Total Human Dose (mg/70kg)	NOAEL and MTD Based on Rat Sedation (Balance Beam Task)
Allo dose (mg/kg)	0.09	0.04	0.02	0.01	**0.5**	
	0.37	0.15	0.07	0.03	**2.0**	
	**0.50**	0.21	0.10	0.04	2.7	
	0.60	0.25	0.12	0.05	3.3	NOAEL Male IV
	0.74	0.31	0.15	0.06	**4.0**	
	**1.00**	0.41	0.20	0.08	5.4	
	1.11	0.46	0.22	0.09	**6.0**	
	1.21	**0.50**	0.24	0.09	6.5	NOAEL Female IV
	**1.50**	0.62	0.30	0.12	8	
	**2.00**	0.83	0.40	0.15	11	
	2.41	1.00	0.48	0.19	13	NOAEL Male IM
	**3.00**	1.24	0.60	0.23	16	
	4.82	**2.00**	0.96	0.37	26	NOAEL Male SC, Female IM; MTD Male and Female IV
	9.65	**4.00**	1.92	0.75	52	NOAEL Female SC
	**10.00**	4.15	1.99	0.77	54	
	15.08	6.25	**3.00**	1.17	82	
	19.29	**8.00**	3.84	1.49	104	MTD Male and Female SC and IM
	25.13	10.42	**5.00**	1.94	136	
	**50.00**	20.74	9.95	3.87	271	

Equivalent dose estimates of allopregnanolone (Allo) for each species assume a comparison of identical formulation and route of administration. Conversion is based on body surface area and computed with the following equation: Human equivalent dose = animal dose (mg/kg) x [animal weight (kg) / human weight (kg)]^0.33^. The exponent 0.33 is an allometric conversion constant [36]. Allo doses indicated in **bold** were administered during the course of the preclinical studies (see Methods section for route of administration and formulation). Total human doses of Allo were calculated in milligrams per 70 kg average body weight. Human doses indicated in **bold** are predicted to be within tolerable range for IV infusion to be tested in a multiple ascending dose trial (Phase 1b clinical trial of Allo for early AD; ClinicalTrials.gov Identifier: NCT02221622 [37]). ***Note*:** *IV doses should not approach or exceed 3 mg/kg rabbit or the equivalent in other species*.

**Table 5:** Estimation of allopregnanolone no observable adverse effect level (NOAEL) and maximally tolerated dose (MTD) in male and female rats and predicted human NOAEL and MTD by multiple routes of administration.

		NOAEL (mg/kg)		MTD (mg/kg)	
Species	Route of Administration	Female	Male	Female	Male
Rat	Subcutaneous	4	2	> 8	8
	Intramuscular	2	< 2	8	< 8
	Intravenous	0.5	< 0.5	2	2
Human (predicted)	Subcutaneous	0.75	0.37	> 1.49	1.49
	Intramuscular	0.37	< 0.37	1.49	< 1.49
	Intravenous	0.09	< 0.09	0.37	0.37

Data from rat sedation studies was used to determine NOAEL and MTD by subcutaneous, intramuscular, and intravenous routes of administration. Allo was administered by bolus push injections within 30 sec via subcutaneous or intramuscular: 6 mg/ml Allo solution in 0.9% sodium chloride with 24% sulfobutyl ether β-cyclodextrin sodium (SBECD); or intravenous: 1.5 mg/ml Allo solution in 0.9% sodium chloride with 6% SBECD. All formulations contained SBECD:Allo molar ratio of 5.89. Sex differences found in rat sedation studies (see Fig 5; Table 3) suggest that males are more sensitive to Allo on the basis of mg/kg body weight.

Collectively, these findings provide translational evidence for a dose range to establish in humans a subsedative dose which would predict neuroregeneration in the targeted populations with mild cognitive impairment or early AD.

## Discussion

### Preclinical pharmacokinetics

In this study, we conducted translational analyses to enable AD clinical trial development. PK analyses, preclinical efficacy, clinical formulation development, and determination of MTD based on sedation as the endpoint were compiled to evaluate Allo. To inform future toxicology studies in non-rodent and rodent species that meet FDA toxicology requirements for therapeutic development, acute bolus dose PK analysis was conducted in female rabbits and male mice. Rabbit and mouse Allo PK profiles after IV administration were similar. Additionally, IV PK parameters from both species (Allo T_1/2_ rabbit 3.3 h; mouse 4 h) were comparable to reported IV human exposure, where the Allo T_1/2_ has been reported to be 4.4 ± 1.7 h [20, 21]. In addition to IV route, the TD route was tested in rabbits. TD Allo was modestly skin-penetrable with 6.8% bioavailability to the plasma and 36% of the plasma-available Allo entered the brain. Although the rabbit IV dose tested was approximately 10-fold higher than the mouse IV dose, when considering differences in species [36], similar results from multiple PK parameters were obtained. After allometric species conversion [36], the IV mouse dose of 1.5 mg/kg was comparable to the Allo dose used in a previous clinical trial, 0.09 mg/kg (see Table 4) [20, 21]. The dosing studies conducted herein combined with the available scientific literature were used to estimate the maximum safe starting dose range [36] for a Phase 1 clinical trial for AD [37].

### Preclinical efficacy

To determine the optimal regimen for therapeutic efficacy of Allo treatment in AD, we investigated the efficacy of acute treatment protocols designed to determine the optimal dose, formulation, and route for Allo treatment to promote the regenerative capacity of the brain. Previously published studies by our group have demonstrated Allo efficacy via the SC route in young 3xTgAD and aged nonTg mice [10, 12, 13]. We established that 3xTgAD mice at 3-months of age and wildtype mice at 15-months have significantly reduced basal levels of hippocampal BrdU+ cells with 15-month wildtype mice exhibiting learning performance deficits comparable to 6-month-old 3xTgAD mice [13]. The age-associated deficits in neurogenesis were rescued by SC Allo treatment [13]. Further, we demonstrated neurogenic efficacy in 3xTgAD mice treated once per week SC Allo 20% HBCD solution for 6 months duration [12]. SC Allo injections have also been effective in inherited developmental neurodegenerative disorder rodent models [38] and the effect of Allo was partly associated with a reduction in oxidative stress [39]. Here, with 5-month-old 3xTgAD mice, prior to formation of extraneuronal plaque, results indicated 24 h SC Allo treatment dose-dependently increased BrdU labeled hippocampal nuclei and PCNA protein expression.

Bridging SC Allo results to IV Allo cyclodextrin-based formulations, we demonstrated dose-dependent neurogenic efficacy of IV Allo for the first time. The activation profile of hippocampal phosphorylated CREB was consistent with the Allo IV pharmacokinetic profile, peaking at 5 min followed by rapid clearance. Interestingly, in a mouse seizure model with the identical formulation and dose used here to demonstrate CREB activation, within 1 min of Allo dose, total protection was found. This response was no longer evident by 15 and 30 min after the IV bolus whereas an IM dose prolonged efficacy for up to 1 h [40, 41]. *In vitro* experiments have demonstrated in cultured Schwann cells treated with 10 nM Allo that by 15 min, cAMP levels more than doubled followed by rapid CREB phosphorylation [42]. The mechanism of Allo-induced neurogenesis takes advantage of the developmentally regulated reversed chloride gradient to activate a calcium-to-CREB signaling cascade to induce mitosis in neural stem cells [5]. In neural stem cells, Allo promotes a rapid, dose-dependent, stereo-specific, and developmentally regulated increase of intracellular calcium concentration via a mechanism that requires both the GABA_A_R and L-type calcium channel [22]. Calcium influx via the voltage-gated L-type calcium channels activates CaMKIV that phosphorylates CREB [43, 44]. Neural progenitor-derived neurons express L-type calcium ion channels within days after cell division and show spontaneous calcium oscillations that support neuronal differentiation [22, 45]. It has been shown that GABA-induced phosphorylation of CREB overlaps with NeuroD expression within one hour [46]. NeuroD increased relative to vehicle at 4 h then decreased by 24 h after IV Allo 1.5 mg/kg. The transient expression of NeuroD is a critical neurodifferentiation element and IV administration of Allo 0.5 mg/kg was efficacious (upregulation of cell cycle proliferation and differentiation markers pCREB, cyclin D2, PCNA, DCX, and NeuroD), well tolerated, and translates to an approximate human dose of Allo 0.04 mg/kg. Characterizing Allo exposure and efficacy after IV administration was used to validate the planned Allo Phase 1b trial design [37], which will utilize the IV route of administration to evaluate safety in humans and will set the tolerable dose level and PK for subsequent efficacy studies.

Allo administered by IM route in 6% SBECD molar ratio 5.89, demonstrated neurogenic efficacy in 5-month-old 3xTgAD mouse hippocampus. IM Allo increased proliferation at 2 mg/kg inducing an increase comparable to SC suspension at 10 mg/kg within 24 h. Both IM 2 mg/kg and SC suspension 10 mg/kg were efficacious, subsedative, and well tolerated.

The formulations tested were intended to evaluate the potential of Allo to be delivered through multiple routes with tailored solvents for each route. Regardless of the route of administration, Allo crosses the blood-brain barrier as evidenced by multiple indicators of target engagement. These include markers of neurogenesis at low doses and profiles of sedation at higher doses. Collectively the data show that route of administration determines dosing with IV requiring the least dose of Allo and TD/IN/SC requiring the highest doses for target engagement. Thus the safety and efficacy profile of Allo can be modified by both dose and route of administration. Clinically, establishing the MTD in a Phase 1 by IV administration is feasible within a restricted dose range. Based on IV data, MTD can be established for alternative routes of administration that may have greater translational feasibility for clinical chronic treatment regimens.

### Formulation development

The complexation ratio of Allo with cyclodextrins such as SBECD is a major determinant of release of Allo into the blood and brain. The cyclodextrin vehicle SBECD does not cross the blood brain barrier [47] but instead facilitates Allo release to steroid carrier proteins in blood. Cyclodextrins represent an optimal vehicle for solubility of Allo in aqueous formulations and SBECD has been tested extensively for safety in animals and humans and is an excipient in several approved drugs [47]. To develop formulations with clinical utility, we tested multiple SBECD:Allo complexation ratios on behavior in adult rats. Rats display more human-like sedation responses compared to mice and thus were used for these analyses. In rats, Allo induces dose-related sleep changes, including a reduced sleep onset latency and an increase in pre-rapid-eye-movement sleep [48]. Allo has a greater tolerance profile than most agonistic modulators of GABA_A_Rs, including benzodiazepine hypnotics [49]. To functionally detect and quantify sedation, a sensitive balance beam motor coordination task was used to determine differences in the delivery and release rate of multiple Allo formulations. Acute motor impairment was used as a surrogate marker, translatable to clinical studies, to assess that Allo reached its target in the CNS/brain.

Soluble SBECD:Allo formulations estimated between 7:1 and 3:1, maximally deliver Allo to systemic circulation resulting in rapid brain uptake observed by altered motor control. In the optimal SBECD:Allo formulation (molar ratio of 5.89), Allo is fully soluble and rapidly bioavailable, indicated by rapid prolonged sedation at the MTD of 8 mg/kg. When the ratio is increased (molar ratio of 23.56), the rate of Allo release into the brain is reduced, indicated by a lack of sedation. Likewise, when the ratio is decreased (molar ratio of 1.47) relative to optimal, the rate of Allo release into the brain was also reduced. The volume of soluble Allo administered in the saturated suspension formulation was limited when dosing 8 mg/kg to rats and results in mild sedative effects likely due to the relatively small fully soluble fraction of Allo, giving the suspension formulation dual properties. Based on these data, it was postulated that the small but fully soluble fraction of Allo was rapidly delivered to the brain in a subfraction dose followed by a slowly absorbed suspension fraction that does not possess the release rate required for sedation at the 8 mg/kg dose level. These pharmacodynamic studies served to identify three distinct formulations that provide rationale for multiple potential release rate options: 1) Slow release 2) Rapid release 3) Rapid release followed by slow release.

### Route of administration and dose-dependent behavior

The sub-sedative (NOAEL) and MTD Allo doses were determined in male and female rats for SC, IM, and IV routes of administration. For clinical translatability, the NOAEL we targeted was chosen to minimize sedation and to ensure an exposure to Allo that was below 50 ng/ml or 157 nmol/L, i.e. equivalent to the blood level measured in normal pregnant women with mild drowsiness during the third trimester [19]. We observed gender differences in the onset and duration of sedation in Allo dose-response studies. Male rats administered 2 mg/kg IV or 8 mg/kg SC/IM displayed the deepest level of sedation. Through SC, IM, and IV routes of administration females administered the same dose as males were less sedated and recovered more rapidly. This sex difference could be explained by the higher number of GABA_A_ receptor binding sites in male brains relative to female brains [50]. Alternatively, a sex difference could be attributed to the observed peak blood levels observed in previous human studies of IV dosing of Allo 0.09 mg/kg with maximum plasma levels of 150 nmol/L (48 ng/ml) in men and 100 nmol/L (32 ng/ml) in women [21].

For therapeutic use of Allo, it was imperative to determine the optimal dose, formulation, and dosing regimen [5]. Weekly exposure to Allo was optimally efficacious in 3xTgAD mice [12] and this regimen will move forward to definitive FDA-compliant chronic toxicology studies in rodent and non-rodent species to meet or exceed the duration of early phase clinical trials [51]. In human phase 1 studies, mild sedation as an indicator of tolerability will establish the MTD. Endogenous Allo levels are increased during stress [52], the menstrual cycle [53], and pregnancy [19]. Use of anti-depressants [54] can also elevate Allo levels whereas depression is associated with lower circulating Allo [55, 56]. Allo levels are reduced in Alzheimer’s disease brains compared to age-matched controls [54, 57]. Currently, the upper physiological Allo blood concentration of 50 ng/ml reached during the third trimester of pregnancy sets the safe boundary for human dosing until chronic toxicology studies conducted under Good Laboratory Practice standards in rodent and non-rodent species are completed and evaluated by regulatory agencies.

Our preclinical results provide useful estimates for the safest starting dose in humans with consideration of the formulation, route of administration, neurogenic efficacy, and tolerability. Keeping the formulation constant, an SC Allo NOAEL dose of 2 mg/kg to rats is equivalent to a human dose of approximately 0.37 mg/kg or ~26 mg for a 70 kg adult (Table 4). Of the formulations tested, Allo 6 mg/ml would require 4.3 ml, which poses a challenge for human SC administration. However, the 24 mg/ml suspension formulation would be a viable option to deliver Allo 26 mg/1.1 ml. For human translation, SC is advantageous due to ease of administration, patient compliance, and tolerability. However, the volume of formulation required to reach the appropriate dose is a limiting factor. Assessed by rat motor coordination test, SC Allo response was more variable than IM Allo response. The data indicated that at high doses with identical formulation, IM route is less variable than SC and approximately twice as potent at induction of sedation, a surrogate indicator of brain uptake. An IM Allo NOAEL dose of 2 mg/kg to mice is equivalent to a human IM dose of approximately 0.15 mg/kg or ~11 mg Allo for a 70 kg adult (Table 4). Translatable to humans, Allo 6 mg/ml in 24% SBECD would require an estimated 2 ml volume IM. PK studies are often conducted via the IV route of exposure to provide data on maximal bioavailability and inform further studies by alternative routes of administration. An IV Allo NOAEL dose of 0.5 mg/kg in mice or 0.21 mg/kg to rats is equivalent to a human IV dose of approximately 0.042 mg/kg or ~3 mg for a 70 kg adult. The vehicle and route of administration were demonstrated to influence PK and pharmacodynamic outcomes and thereby the therapeutic range of Allo.

## Conclusions

PK, pharmacological efficacy, and behavioral studies were conducted to inform Allo formulation development, safety monitoring, and pharmacokinetic interpretation in clinical trials (Phase 1b clinical trial of Allo for early AD; ClinicalTrials.gov Identifier: NCT02221622 [37]). Results of the rabbit and mouse PK studies, demonstrated that soluble cyclodextrin-based bolus IV injections rapidly delivered Allo to blood and brain and reached peak levels in brain within 5min which was then rapidly cleared. While the rabbit PK dose was at MTD level, the mouse PK dose was near NOAEL and was accompanied by indicators of efficacy in hippocampal neurogenesis indicating that efficacy can be achieved within safe dosing limits. In mouse, the suspension SC Allo peak levels were much lower than IV with slower uptake to the brain. Data from our studies demonstrate that Allo SC, administered in cyclodextrin-solublized form, and IV Allo were efficacious at NOAEL doses in both aged wildtype mice and young 3xTgAD mice. The dose-responses in these studies indicated that lower sub-sedative doses were optimal for both efficacy and safety. The dose response curve for Allo exhibits an inverted U-shaped function for neuroregeneration where lower doses are on the ascending arm of the dose response curve whereas higher doses are on the descending arm [5, 10–12]. The rat behavior studies allowed for formulation development and were used to compare sedation responses as a surrogate detection method to brain Allo PK across different routes of administration. The IV route is standard for early phase safety and PK clinical trials and control of IV infusion rate to mimic the IM or SC delivery rate of Allo can be achieved. Other options explored here, the TD and IN routes are less-invasive alternatives that may prove to be most clinically translatable. In cyclodextrin-based formulations, oral Allo bioavailability is generally not sufficient although food oils may enhance solubility for the treatment of medical conditions [58]. Previous human trials with IV Allo were formulated in albumin and tested in young adults [21]. Clinical dose-escalating studies with a cyclodextrin-based Allo formulation in elderly people are warranted to determine safety and tolerability for use in early AD and other unmet neurological disorders [2, 5, 59]. Collectively, the translational research described herein build upon our previous preclinical results in relevant animal models [10, 12, 13] to provide key insights that predict the maximally safe starting dose for humans, efficacy by several routes of administration, and the optimal Allo formulation complex necessary to advance to proof of concept clinical studies of Allo as a regenerative and disease modifying therapeutic for Alzheimer’s disease.
