# Correction: Allopregnanolone Preclinical Acute Pharmacokinetic and Pharmacodynamic Studies to Predict Tolerability and Efficacy for Alzheimer's Disease

**DOI:** 10.1371/journal.pone.0132210

**Published:** 2015-06-29

**Authors:** The PLOS ONE Staff

In the published article Fig 3 is missing panels I-N. Please view the complete, correct, Fig 3 here.

**Fig 3:**
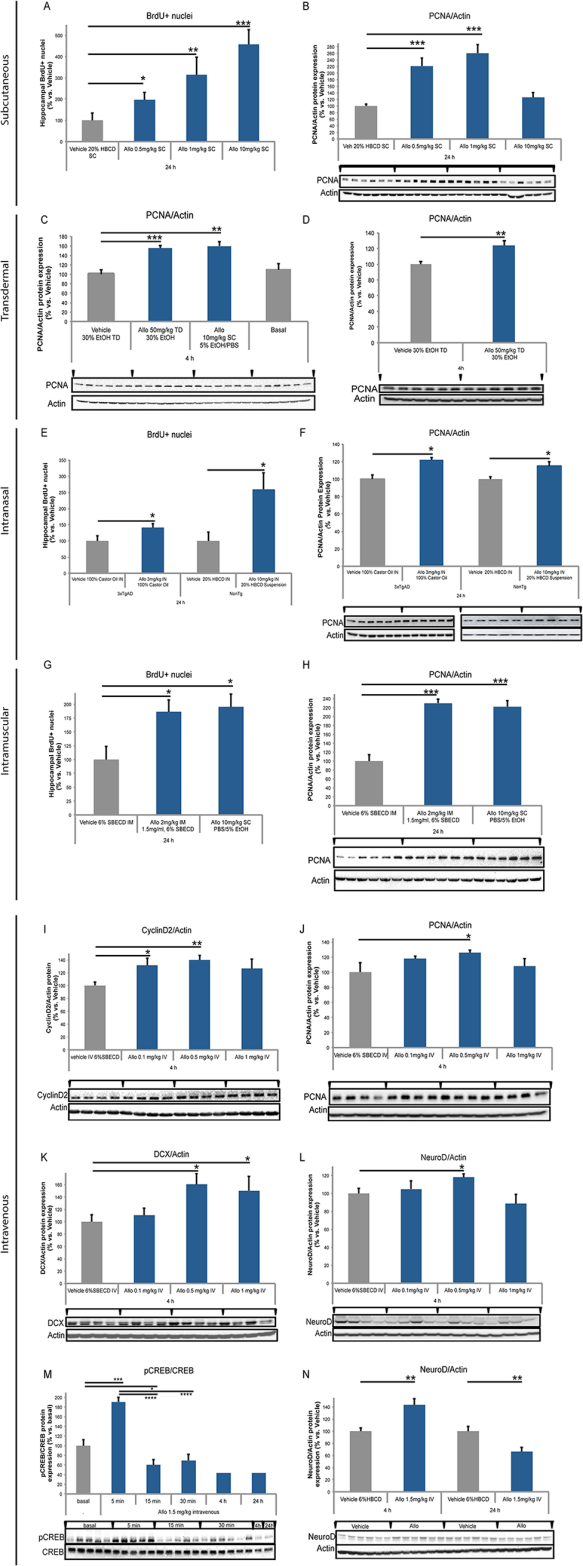
Subcutaneous Allo increased BrdU incorporation and PCNA protein expression in male mouse AD model. A. In 5-month-old 3xTgAD mouse hippocampus, BrdU+ nuclei increased significantly in 24 h after a single subcutaneous (SC) dose of Allo 0.5, 1, or 10 mg/kg. B. In 5-month-old 3xTgAD mouse hippocampus, protein expression of ~29 kDa PCNA increased significantly at 24 h after SC Allo 0.5 and 1 mg/kg doses whereas 10 mg/kg dose trended towards increase but did not reach significance. Transdermal and subcutaneous Allo increased PCNA protein expression in male mouse AD model. C. In 5-month-old 3xTgAD mouse hippocampus, protein expression of PCNA increased significantly at 4 h after transdermal (TD) Allo 50 mg/kg and SC Allo 10 mg/kg doses. D. In 15-month-old nonTg mouse hippocampus, protein expression of PCNA increased significantly at 4 h after TD Allo 50 mg/kg dose. E. In 5-month-old 3xTAD and 15-month-old nonTg mouse hippocampus, BrdU+ nuclei increased significantly in 24 h after intranasal (IN) dose of Allo 3 mg/kg 100% Castor Oil and Allo 10 mg/kg 20% HBCD suspension doses. F. In 5-month-old 3xTgAD and 15-month-old nonTg mouse hippocampus, protein expression of PCNA increased significantly at 24 h after IN Allo 3 mg/kg 100% Castor Oil and Allo 10 mg/kg 20% HBCD suspension doses. Intramuscular Allo-induced increase in cell cycle marker in male mouse AD model. G. In 5-month-old 3xTgAD mouse hippocampus, BrdU+ nuclei increased significantly in 24 h after a single intramuscular (IM) dose of Allo 2 mg/kg and SC Allo 10 mg/kg dose. H. In 5-month-old 3xTgAD mouse hippocampus, protein expression of PCNA increased significantly at 24 h post-IM Allo 2 mg/kg dose and SC Allo 10 mg/kg dose. Intravenous Allo-induced increase in cell cycle and neurodifferentiation markers in male mouse AD model. I. In 5-month-old 3xTgAD mouse hippocampus, protein expression of 30 kDa cyclinD2 increased significantly at 4 h post-intravenous (IV) Allo 0.1 and 0.5 mg/kg dose whereas 1 mg/kg dose trended towards increase but did not reach significance. J. In 5-month-old 3xTgAD mouse hippocampus, protein expression of PCNA increased significantly at 4 h after IV Allo 0.5 mg/kg dose whereas 0.1 and 1 mg/kg dose did not reach significance. K. In 5-month-old 3xTgAD mouse hippocampus, protein expression of ~40 kDa doublecortin (DCX) increased significantly at 4 h after IV Allo 0.5 and 1 mg/kg doses. L. In 5-month-old 3xTgAD mouse hippocampus, protein expression of 49 kDa NeuroD increased significantly at 4 h after IV Allo 0.5 mg/kg, whereas 0.1 and 1 mg doses did not reach significance. Intravenous Allo-induced rapid transient increase in CREB phosphorylation in male mouse aging model. M. In 15-month-old nonTg mouse hippocampus, protein expression of 43 kDa serine 133 phosphorylated CREB (pCREB) increased significantly 5 min after IV Allo 1.5 mg/kg dose. N. In 15-month-old nonTg mouse hippocampus, protein expression of 49 kDa NeuroD1 (NeuroD) increased significantly at 4 h then decreased at 24 h after intravenous Allo 1.5 mg/kg dose. * p<0.05, ** p<0.01, *** p<0.001, **** p<0.0001, *n* = 4–6, bars represent mean value ± SEM.
